# Leptin induces upregulation of sphingosine kinase 1 in oestrogen receptor-negative breast cancer via Src family kinase-mediated, janus kinase 2-independent pathway

**DOI:** 10.1186/s13058-014-0426-6

**Published:** 2014-10-25

**Authors:** Heba Alshaker, Jonathan Krell, Adam E Frampton, Jonathan Waxman, Oleg Blyuss, Alexey Zaikin, Mathias Winkler, Justin Stebbing, Ernesto Yagüe, Dmitri Pchejetski

**Affiliations:** 1 Department of Surgery and Cancer, Imperial College London, 1st Floor ICTEM, Hammersmith Hospital, Ducane Road, London, W120NN UK; 2 Department of Pharmacology and Biomedical Sciences, Faculty of Pharmacy and Medical Sciences, Petra University, Amman, Jordan; 3 Institute for Women's Health, University College London, 74, Huntley Street, London, WC1E 6AU UK; 4 School of Medicine, University of East Anglia, Elizabeth Fry Building, Norwich Research Park, Norwich, NR47TJ UK

## Abstract

**Introduction:**

Obesity is a known risk factor for breast cancer. Sphingosine kinase 1 (SK1) is an oncogenic lipid kinase that is overexpressed in breast tumours and linked with poor prognosis, however, its role in obesity-driven breast cancer was never elucidated.

**Methods:**

Human primary and secondary breast cancer tissues were analysed for SK1 and leptin receptor expression using quantitative real-time polymerase chain reaction (qRT-PCR) assay. Leptin-induced signalling was analysed in human oestrogen receptor (ER)-positive and negative breast cancer cells using Western blotting, qRT-PCR and radiolabelling assays.

**Results:**

Our findings show for the first time that human primary breast tumours and associated lymph node metastases exhibit a strong correlation between SK1 and leptin receptor expression (Pearson *R* = 0.78 and *R* = 0.77, respectively, *P* <0.001). Both these genes are elevated in metastases of ER-negative patients and show a significant increase in patients with higher body mass index (BMI). Leptin induces SK1 expression and activation in ER-negative breast cancer cell lines MDAMB-231 and BT-549, but not in ER-positive cell lines. Pharmacological inhibition and gene knockdown showed that leptin-induced SK1 activity and expression are mediated by activation of extracellular signal-regulated kinases 1/2 (ERK1/2) and Src family kinase (SFK) pathways, but not by the major pathways downstream of leptin receptor (LEPR) - janus kinase 2 (JAK2) and signal transducer and activator of transcription 3 (STAT3). Src-homology 2 domain-containing phosphatase 2 (SHP2) appeared to be key to SK1 activation, and may function as an adaptor protein between SFKs and LEPR. Importantly, leptin-induced breast cancer cell proliferation was abrogated by SK1-specific small interfering RNA (siRNA).

**Conclusions:**

Overall, our findings demonstrate a novel SFK/ERK1/2-mediated pathway that links leptin signalling and expression of oncogenic enzyme SK1 in breast tumours and suggest the potential significance of this pathway in ER-negative breast cancer.

**Electronic supplementary material:**

The online version of this article (doi:10.1186/s13058-014-0426-6) contains supplementary material, which is available to authorized users.

## Introduction

Overall 33% of the world’s adult population are overweight or obese and, if this trend continues, by 2030 this figure will be doubled [1]. Obesity is a risk factor for poor breast cancer prognosis [2] and metastasis [3]; and oestrogen production and adipokine secretion were tagged as key elements in this relationship [2]-[4]. Leptin is one of the prominent adipokines and its intratumoural levels are positively correlated with poor breast cancer prognosis [5], advanced stage [3], metastasis and recurrence [6]. The critical role of leptin (rather than just obesity) in breast cancer progression was highlighted in an elegant experiment where obese *ob/ob* mice, which lack leptin, showed reduced mammary tumour outgrowth compared to increased tumour growth in obese *db/db* mice, lacking functional leptin receptor (LEPR) and hence having high circulating leptin levels [7]. LEPR is highly expressed in breast tumour tissue and has six splice variants encoding four isoforms that share an identical intracellular domain Box1 that is critical for Janus kinase (JAK) binding and activation. The longest isoform of LEPR (LEPR-Long) also contains a binding site for signal transducer and activator of transcription (STAT) [8]. Leptin signalling triggers activation of extracellular signal-regulated kinases 1/2 (ERK1/2), STAT3, and phosphatidylinositol 3-kinase (PI3K)/Akt [9]. Interestingly, leptin retains the ability to stimulate STAT3 and ERK1/2 in cells lacking JAK2 kinase, where JAK2-independent responses appear to be mediated by members of the src family kinases (SFKs) [10].

Leptin promotes cellular proliferation in both oestrogen receptor (ER)-positive and -negative breast cancer cell lines [11]. In breast cancer leptin is also a positive regulator of vascular endothelial growth factor (VEGF) and blockade of leptin signalling markedly reduces VEGF expression and the tumour growth in mouse xenografts [12].

Sphingosine kinase 1 (SK1) is an oncogenic enzyme that is highly expressed in human tumours and has been shown to act as a `signalling hub’ mediating cancer progression, angiogenesis and cell migration, making it a key molecule in the search for potential anticancer therapies [13]. High levels of SK1 expression have been shown in various human tumour tissues (including breast) [14], where they enhance angiogenesis and are associated with chemoresistance and a poor prognosis [15]. SK1 mRNA expression increases through the four stages of breast cancer [16], is higher in ER-negative tumours and is associated with disease progression and poor prognosis [17], however, the mechanism of its upregulation is not determined.

The metabolic profile of fat tissue from obese subjects (compared with lean subjects) exhibits a high content of sphingosine-1-phosphate (S1P, a product of SK1 activity), which promotes proliferative responses, suggesting that obesity may be a factor affecting SK1 levels [18]. Until now no direct links between leptin-mediated signalling and SK1 in breast cancer have been documented.

Our data show for the first time a strong correlation between SK1 and LEPR expression in human primary breast tumours and associated lymph node metastases. The levels of both genes were significantly increased in patients with higher body mass index (BMI) and in the lymph node metastases of ER-negative patients. In ER-negative breast cancer cells leptin induces SK1 expression and activation through ERK1/2 and src-homology 2 domain-containing phosphatase 2 (SHP2)/SFKs pathways and independently of JAK2/STAT3. Consequently, SK1 activation is critical for leptin-induced breast cancer cell proliferation. Our findings demonstrate a novel pathway that links leptin signalling and expression of oncogenic enzyme SK1 in breast tumours, which may have a physiological significance in obesity driven ER-negative breast cancer.

## Materials and methods

### Patients' samples

Archival paraffin-embedded tissue from 69 patients with primary breast tumours and corresponding lymph node (LN) metastases was obtained from Imperial College NHS Trust tissue bank. Prior to donating their samples to the bank, all patients consented for their subsequent use in research projects and publications. The study was approved by National Research Ethics Service and performed in accordance with ethical guidelines. None of the patients received neo-adjuvant therapy. Clinicopathological details of the patients enrolled in this study are listed in Table S1 in Additional file 1. All samples were formalin-fixed and paraffin-embedded (FFPE). Four sections (5 μm thick) were macrodissected from the FFPE blocks with trimming of excess paraffin. Only tissue containing at least 70% of tumour was used for RNA isolation.

### Chemicals and antibodies

Recombinant human leptin was obtained from Sigma-Aldrich (St. Louis, MO, USA). [γ-^32^P]-ATP (6,000 mCi/mmol) was purchased from PerkinElmer (Waltham, MA, USA), silica gel G60 plates from GE Healthcare (Waukesha, WI, USA) and sphingosine from Avanti Polar Lipids (Alabaster, AL, USA). Wortmannin, LY294002, and SU6656 were from Calbiochem (Darmstadt, Germany), UO126 was from New England Biolabs (Hitchin, UK). Antibodies for p-SFK (Tyr416), total Src (cross-reacts with other SFK family members), ERK 1/2, phospho (p)-STAT 3, p-Akt (Ser473), Akt, JAK2 were obtained from New England Biolabs (Hitchin, UK). p-ERK 1/2 from Sigma-Aldrich (Steinhelm, Germany), STAT-3 from Santa Cruz Biotechnology (Heidelberg, Germany). Other reagents and chemicals used were purchased from Sigma-Aldrich (Gillingham, UK) unless otherwise specified.

### Cell culture

Breast cancer cell lines MDAMB-231, BT-549, MCF7 and BT-474 were purchased from ATCC (Manassas, VA, USA). All cells were maintained in tissue culture flasks or plastic dishes in Dulbecco’s modified Eagle’s medium (DMEM) with 10% heat-inactivated foetal calf serum (FCS) (FirstLink, Birmingham, UK), 50 U/ml penicillin, 50 μg/ml streptomycin and 2 mM glutamine (Sigma-Aldrich, St. Louis, MO, USA) in a humidified atmosphere of 5% CO_2_ at 37°C. Cell lines were kept in culture for up to 30 passages.

### Cell treatment and preparation of cell lysates

Cells were seeded to reach 70 to 80% confluence by the end of the treatment. Next day, cells were washed with serum-free media and starved overnight. Leptin and pharmacological inhibitors (1 h prior to leptin) were added at the concentrations and for the times indicated in the figure legends. After incubation cells were washed with ice-cold phosphate-buffered saline (PBS) and then harvested.

### RNA extraction and cDNA synthesis and qRT-PCR

Samples were dissolved in RLT lysis buffer and isolation of total RNA was performed using the RNeasy Mini kit (Qiagen, Valencia, CA, USA) as per manufacturer’s instructions. RNA quantity and purity was measured using a NanoDrop ND-100 Spectrophotometer (Thermo Fisher Scientific, Loughborough, UK). Reverse transcription was performed using Precision nanoScript™ Reverse transcription kit (PrimerDesign Ltd, Southampton, UK). qRT-PCR was done using Precision™ 2X qPCR Mastermix with SYBR green™ and 6-carboxyl-X-rhodamine (ROX) as a reference dye (PrimerDesign Ltd, Southampton, UK) as per manufacturer's instructions. For measurement of breast cancer clinical samples, double-dye (Taqman™) primers (Table S2 in Additional file 1) were used with PrimerDesign 2X Precision™ MasterMix containing ROX using ABI PRISM 7900 sequence detection system (Applied Biosystems, Darmstadt, Germany). Ct values were exported and analysed using qbase software (Biogazelle NV, Zwijnaarde, Belgium) using multiple reference genes normalisation. The expression of SYBR green target genes (SK1, VEGF and SHP2) was normalised to three reference genes: ubiquitin C (UBC), glyceraldehyde-3-phosphate dehydrogenase (GAPDH), and tyrosine-3-monooxy-genase/tryptophan 5-mono-oxygenase activation protein (YWAZH). Breast cancer clinical samples were normalised to five housekeeping genes: GAPDH, beta glucuronidase (GUSB), TATA box binding protein (TBP), eukaryotic 18S rRNA (18S) and mitochondrial ribosomal protein L19 (MRPL19).

### Proliferation assay

Cells were seeded in 96-well plates and incubated for 24 h, then starved for 24 h and then incubated with or without leptin and/or SK1 small interfering RNA (siRNA). MTT assay - 5 mg/ml 3-(4,5-dimethylthiazol-2-yl)-2,5-diphenyl tetrazolium bromide (MTT) was added to each well. After 3.5 h of incubation at 37°C, supernatant was aspirated and formazan crystals were dissolved in 0.5 M dimethylformamide and 20% SDS. Optical density was read at 570 nm using a microplate reader (Tecan Sunrise™, Mannedorf, Switzerland).

### Western blotting

Western blot analysis was performed as previously described [19],[20]. Cell pellets were dissolved in 1X SDS loading dye and protein quantification was carried out by Dc protein assay (Bio-Rad Laboratories, Hercules, CA, USA). Proteins (20 to 30 μg) were loaded on acrylamide mini gels and run at 110 V. Separated proteins were transferred onto PVDF Immobilon-P™ membranes then blocked in PBS-T containing 5% (w/v) non-fat dry milk for 1 h at room temperature. Primary antibodies were diluted in 5% bovine serum albumin (BSA)/PBS-T containing sodium azide and incubated overnight at 4°C. Secondary peroxidase-conjugated antibodies against mouse or rabbit IgG (GE Healthcare, Amersham, UK) were added in PBS-T/milk. Membranes were exposed to chemiluminescent horseradish peroxidase (HRP) substrate (GE Healthcare (Amersham, UK)) and visualised using X-ray films (SLS, Hessle, UK) on an SRX-101A X-ray developer.

### Sphingosine kinase assay

SK1 assay was performed using radiolabelling as previously described [21]. Cell lysates were resuspended in SK1 buffer (20 mM Tris-HCl pH7.4, 20% glycerol, 1 mM 2-mercaptoethanol, 1 mM EDTA, 10 μg/ml PMSF, 15 mM NaF, 10 μg/ml leupeptin, aprotinine, Soybean trypsin inhibitor, 0.5 mM 4-deoxypyridoxine, 40 mM B-glycerophosphate, 1 mM sodium orthovanadate). Lysates were sonicated and centrifuged at 20,000 g for 30 min at 4°C. Protein concentration in the supernatant was estimated by Bradford protein assay (Bio-Rad, Munich, Germany). Each sample was resuspended in 200 μl SK1 buffer and 50 mM sphingosine, 20 mM MgCl_2,_ 20 mM ATP and 10 μCi [γ-^32^P]-ATP (6000 Ci/mmol) were added and incubated for 1 h at 37°C. The reaction was stopped by addition of 50 μl 1 M HCl, 800 μl chloroform/methanol/HCl, 240 μl chloroform and 240 μl 2 M KCL. After centrifugation, the lower organic phase was collected and vaporised. Dried lipids were solubilised with 40 μl chloroform/methanol (2:1, v/v) and separated by thin layer chromatography on silica gel G60 plates using 1-butanol/ethanol/acetic acid/water (80:20:10:20, v/v) as migration solution. Plates were air-dried, exposed to X-ray film and quantified using Image J software.

### RNA interference

Cells were seeded at a density to reach 30 to 50% confluence by the day of transfection. Forty nm siRNA oligonucleotides combined with oligofectamine in Opti-MEM™ (Invitrogen, Carlsbad, CA, USA) were used. siRNA directed against SK1 was obtained from Applied Biosystems. All other siRNAs were purchased from Thermo Fisher Scientific (Loughborough, UK) as pooled four independent sequences. Non-targeting siRNA, were used as a negative control (Table S3 in Additional file 1). Optimal knockdown was obtained 72 h post-transfection and verified by western blot or qRT-PCR.

### Statistical analysis

Data are presented as the mean values normalised to control ± standard error of the mean (SEM) calculated using GraphPad Prism (GraphPad Software, La Jolla, CA, USA). Statistical significance between two groups was conducted by unpaired Student’s *t* test. Comparisons between the means of more than two groups were assessed using one-way ANOVA analysis followed by a Tukey’s test (95% confidence). The Pearson’s correlation coefficient was calculated between the expression levels of two target genes. Multivariate analysis was performed to find the parameters fitting the best linear regression model based on the minimization of Akaike information criterion. The odds ratio (OR) for all pairs of parameters has been calculated via logistic regression.

## Results

### Expression of LEPR-Long and SKcorrelates in human breast tumours and metastatic lymph nodes, and is elevated in patients with high BMI

**Figure 1:**
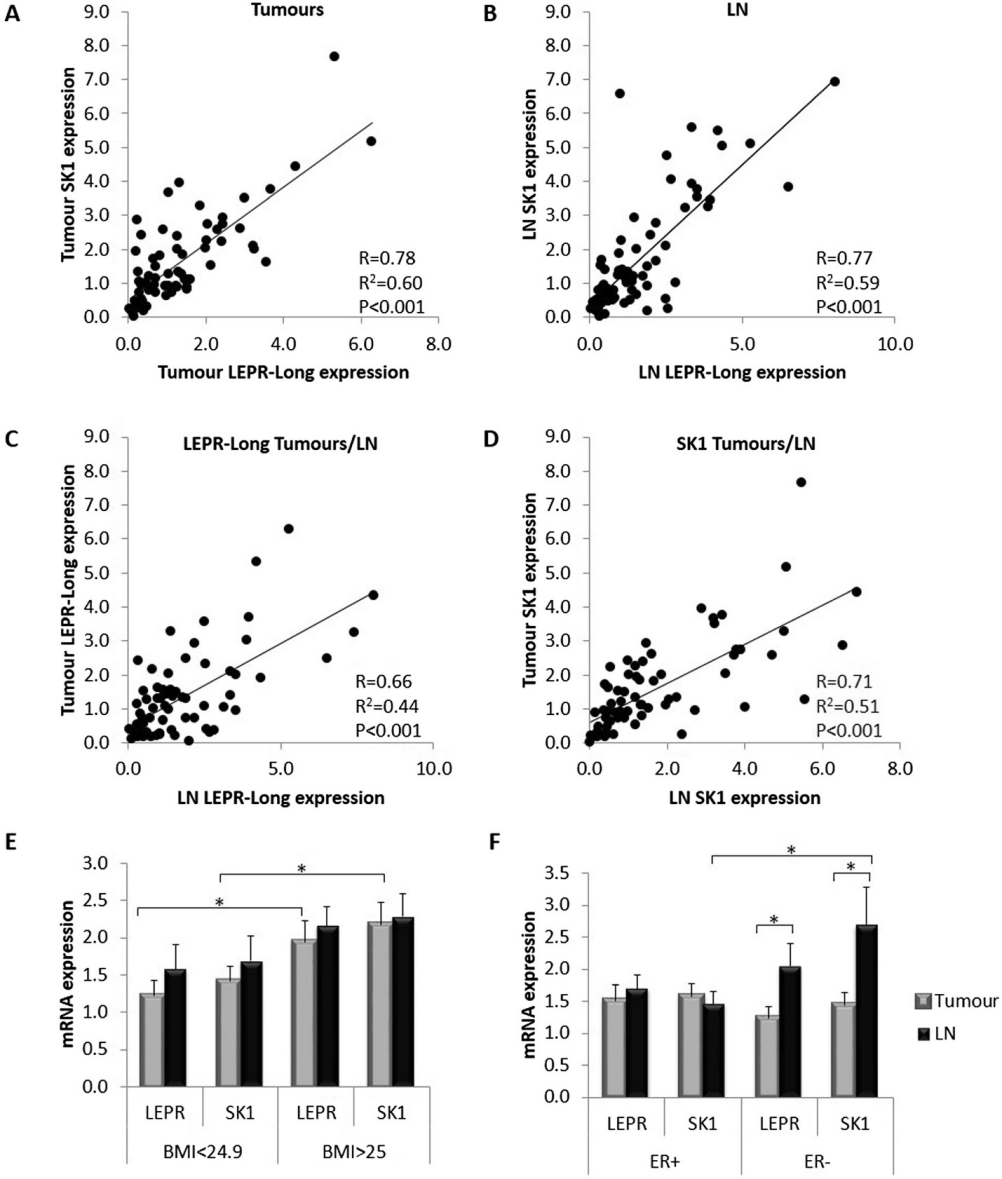
**SK1 expression correlates with LEPR-Long expression in tumours and positive lymph nodes from breast cancer patients with higher expression of those genes oestrogen receptor-negative breast lymph-node metastases and in the tumours of obese and overweight patients.** RNA was extracted from human breast cancer samples and expression of SK1 and LEPR-Long mRNAs was determined by qRT-PCR, (normalised against GAPDH, GUSB, TBP, 18S and MRPL19) and analysed using qBase software. Correlation between SK1 and LEPR-Long expression in primary tumours **(A)** and metastatic LNs **(B)**. Correlation between LEPR-Long **(C)** and SK1 **(D)** expression in primary tumours and metastatic LN. **(E)** Expression of LEPR-Long and SK1 with respect to BMI (<24.9 or >25) in primary tumours and metastatic LN. **(F)** Expression of LEPR-Long and SK1 in primary tumours and metastatic LNs of ER-positive and -negative breast cancer patients. Pearson correlation coefficient (R), square Pearson correlation coefficient (R^2^) and statistical significance (*P*) are indicated. Columns*,* represent the mean; bars*,* SEM. (*, *P* <0.05; **, *P* <0.01; §, *P* <0.001; NS, not significant, *P* >0.05). ER, oestrogen receptor; LNs, lymph nodes.

We have used Taqman qRT-PCR to quantify LEPR-Long and SK1 expression in macrodissected paraffinised sections of breast tumours and corresponding LN metastases obtained from 69 patients. There was a strong positive correlation between LEPR-Long and SK1 both in tumours (*P* <0.001, R = 0.78) and LNs (*P* <0.001, R = 0.77) (Figure 1A,B). Both LEPR-Long and SK1 expression in primary tumours correlated with their relative expression in corresponding metastatic LNs (Figure 1C,D). Importantly, patients with higher BMI had higher levels of LEPR-Long and SK1 in tumours (*P* <0.05) and LNs (not significant) (Figure 1E). Furthermore, in ER-negative patients, SK1 and LEPR-Long expression was significantly higher in metastatic LNs than in primary tumours or LNs from ER-positive patients (Figure 1F). A similar trend was noticed, albeit to a lower extent, in triple-negative tumours when compared to triple-positive (Figure S1A in Additional file 2). Analysis of LEPR-Long and SK1 expression with respect to progesterone receptor (PR), and human epidermal growth factor receptor (HER) expression and menopausal status in tumours and LNs showed no significant differences (Figure S1B-D in Additional file 2). Multivariate linear regression model for outcomes (LN LEPR-Tumour LEPR) and (LN SK1-Tumour SK1) based on the minimization of Akaike information criterion showed that combination of ER status and tumour size had the best correlation to the LEPR and SK1 expression (Table S4 in Additional file 1). To estimate the pairwise influence of parameters, we have split them by tertiles and calculated odds ratios (ORs) with respect to the first group via a logistic regression (Tables S5,S6 in Additional file 1). The combination of tumour grade and HER2 status showed the most significant ORs of (9.8 and 4.1) to have LEPR expression higher in lymph nodes than in tumours (Table S5 in Additional file 1). Table S6 in Additional file 1 shows that the risk for high SK1 expression is elevated in ER-negative (OR = 9.9), or obese (OR = 9.625) patients, or both (OR = 6.111).

### Leptin induces breast cancer cell proliferation

**Figure 2:**
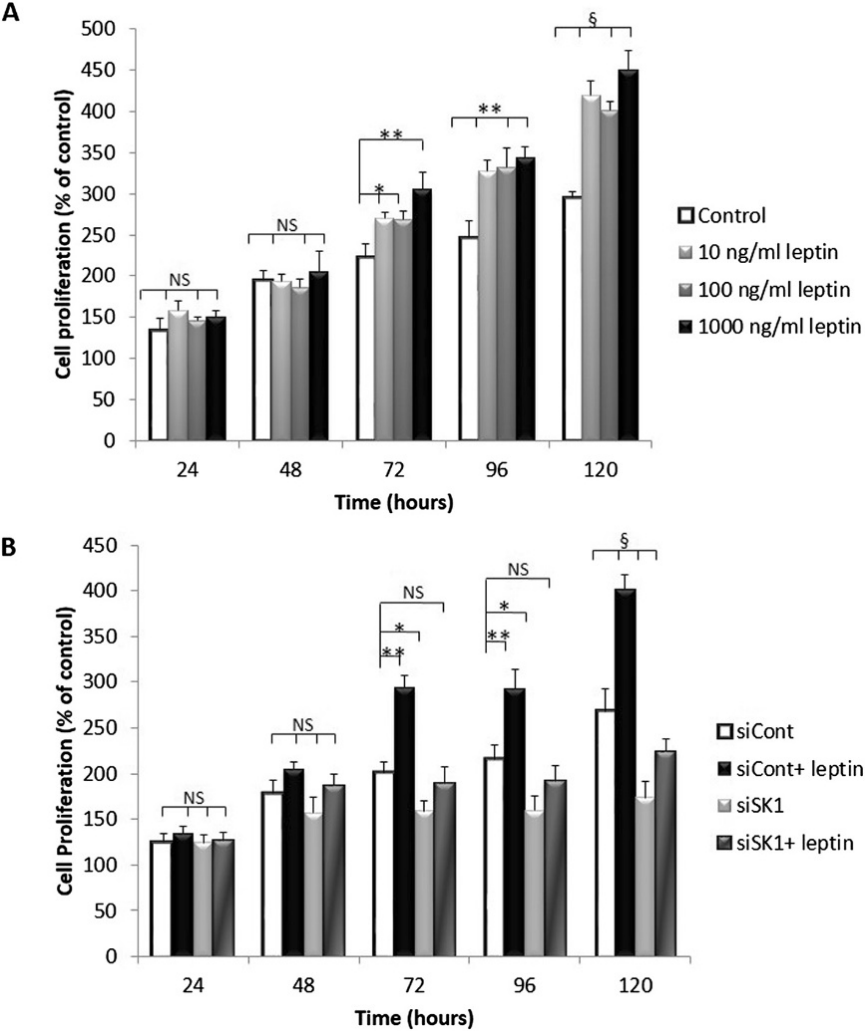
**Leptin does not increase the proliferation of MDAMB-231 cells in the absence of SK1 signalling. (A)** MDAMB-231 cells were starved overnight then incubated with 10 to 1,000 ng/ml leptin for 5 days. Cell proliferation was followed using MTT assay. **(B)** MDAMB-231 cells were transfected with specific siRNA against SK1 (siSK1) or control siRNA (siCont). Cells then were starved overnight then incubated with 1,000 ng/ml leptin for 5 days. Cell proliferation was followed using MTT assay. Columns*,* mean of three independent experiments performed in sextuplicate; bars*,* SEM. (*, *P* <0.05; **, *P* <0.01; §, *P* <0.001; NS, not significant, *P* >0.05).

Assessment of cellular proliferation by MTT assay showed that MDAMB-231 cells treated with 10, 100 and 1,000 ng/ml leptin exhibited a significant increase in proliferation from 72 to 120 h, with the maximum effect obtained at 1,000 ng/ml (Figure 2A). Importantly, specific knockdown of SK1 has abolished these proliferative responses in both MDAMB-231 and BT-549 cells (Figure 2B; Figure S2 in Additional file 2).

### Analysis of leptin-induced pathways in ER-negative and -positive breast cancer cell lines

**Figure 3:**
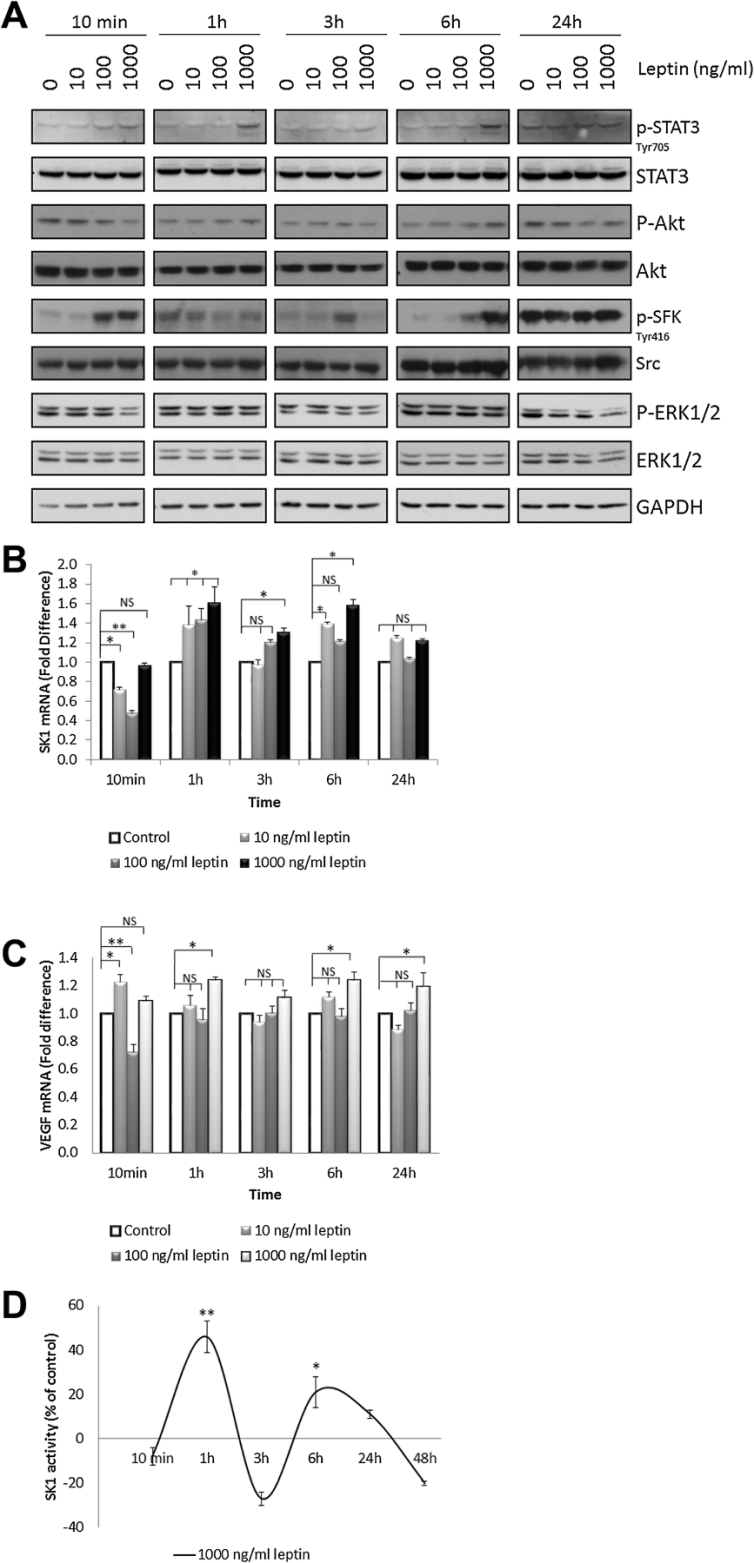
**Leptin activates p-STAT3 and P-SFK, increases SK1 expression and enzymatic activity and VEGF expression in MDAMB-231 cells.** Cells were starved overnight in serum-free media then exposed to 10 to 1,000 ng/ml of leptin for indicated times. **(A)** Cell lysates obtained after each time point were separated on a 10% SDS-PAGE gel and probed for phosphorylation of STAT3, Akt, SFK and ERK1/2. Blots are representative of three independent experiments. Expression of SK1 **(B)** and VEGF **(C)** determined by qRT-PCR, normalised against housekeeping genes (GAPDH, YWHAZ and UBC) and analysed using qBase software. **(D)** SK1 activity was measured by radiolabelling of sphingosine in cell lysates containing equal amounts of protein. Columns, mean of three independent experiments; bars, SEM. (*, *P* <0.05; **, *P* <0.01; §, *P* <0.001; NS, not significant, *P* >0.05).

Dose-dependent treatment of MDAMB-231 cells with leptin showed that 1,000 ng/ml led to a maximal STAT3 phosphorylation at 1 h and 6 h, and to a lesser extent at 10 min. This was paralleled by a marked increase in SFKs phosphorylation (*P* <0.001) (Figure 3A; Figure S3 n Additional file 2). Importantly, 1,000 ng/ml leptin induced a 30 to 60% increase in SK1 mRNA expression at 1 to 6 h, which was mirrored by VEGF (Figure 3B,C). Similarly to mRNA expression, 1,000 ng/ml leptin triggered a 46% increase in SK1 activity after 1 h of treatment (*P* <0.01) followed by a second peak at 6 h (26% increase, *P* <0.05) (Figure 3D). Another ER-negative breast cancer cell line BT-549 showed a similar response (Figure S4,S5 in Additional file 2). In ER-positive MCF-7 and BT-474 cells leptin induced a marked increase in STAT3 phosphorylation, but failed to induce SFK activation or SK1 and VEGF expression (Figure S6-S8 in Additional file 2), indicating the prevalence of this pathway in ER-negative cells.

### JAK2/STAT3 and PI3K/Akt pathways regulate leptin-induced expression of VEGF, but not of SK1

**Figure 4:**
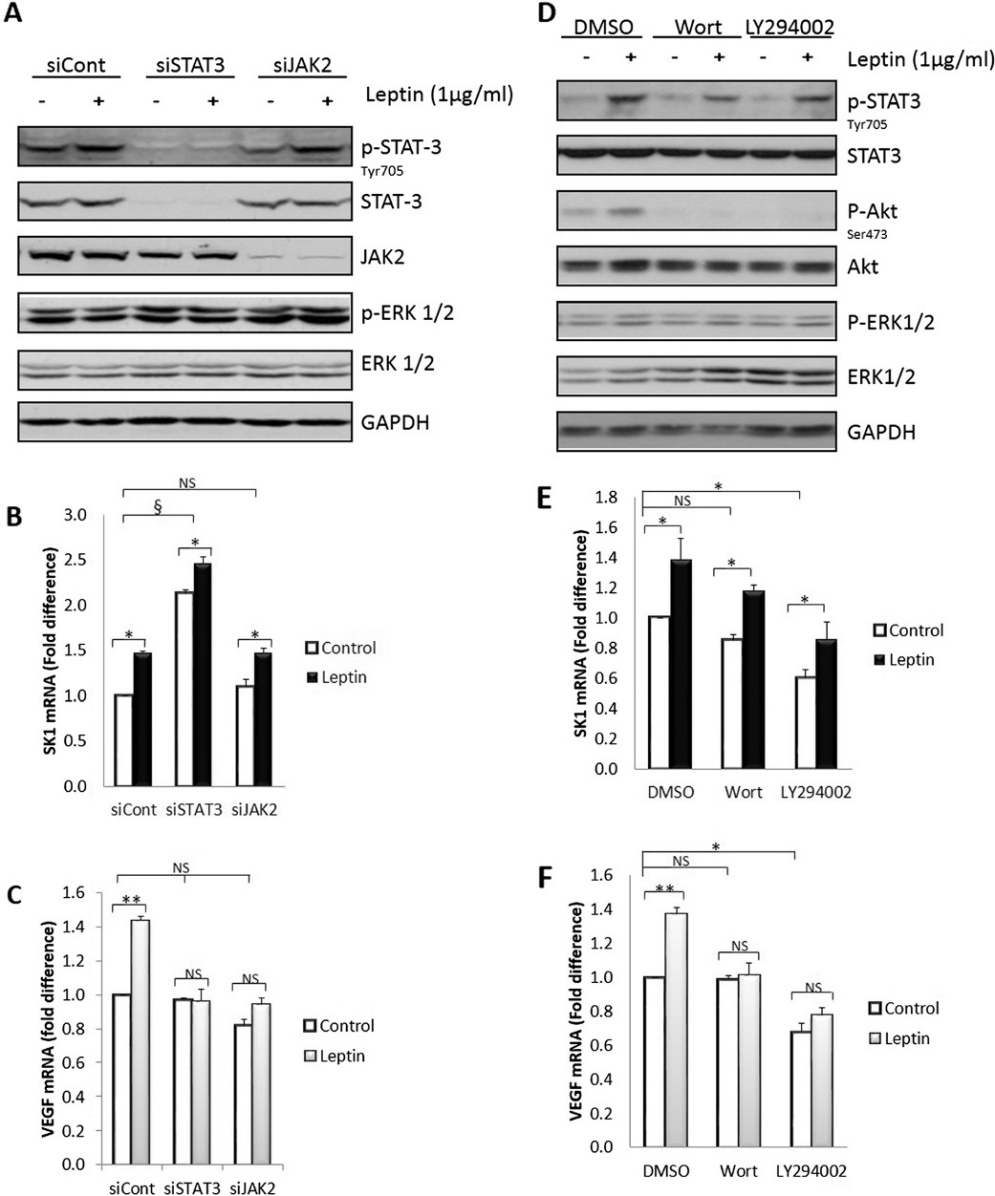
**JAK2 silencing or PI3K/Akt inhibition does not abrogate SK1 expression. (A-C)** MDAMB-231 cells were transfected with specific siRNA against STAT3 (siSTAT3) and JAK2 (siJAK2) or control siRNA (siCont). Cells were starved overnight in serum-free media then exposed to 1,000 ng/ml leptin for 6 h. **(D-F)** MDAMB-231 cells were starved overnight in serum-free media then pre-treated with PI3K/Akt inhibitors wortmannin (1 μM) and LY294002 (10 μM) for 1 h followed by stimulation with 1,000 ng/ml leptin for 6 h. **(A, D)** Cell lysates obtained were separated on a 10% SDS-PAGE gel and probed for phosphorylation of STAT3, JAK2 and ERK1/2 to verify knockdown efficiency or Akt to confirm that the inhibitor worked under the experimental conditions. Expression of SK1 **(B, E)** and VEGF **(C, F)** determined by qRT-PCR, normalised against housekeeping genes (GAPDH, YWHAZ and UBC) and analysed using qBase software. Columns, mean of three independent experiments performed in triplicate; bars, SEM. (*, *P* <0.05; **, *P* <0.01; §, *P* <0.001; NS, not significant, *P* >0.05) when comparing levels of SK1, and VEGF mRNA to siCont levels.

Binding of leptin to its receptor LEPR-Long autophosphorylates JAK2, which in turn activates the tyrosine residue leading to STAT3 phosphorylation [9]. Of note, STAT3 phosphorylation was only modestly decreased upon JAK2 silencing (Figure 4A; Figure S9A in Additional file 2). Likewise, silencing of JAK2 or STAT3 had no effect on leptin-induced SK1 expression (Figure 4B), while leptin-induced VEGF expression was abolished (Figure 4C). Surprisingly, knockdown of STAT3 dramatically upregulated SK1 expression (*P* <0.001) in MDAMB-231 (Figure 4B) and BT-549 cells (Figure S10 in Additional file 2). To exclude an `off-target' effect of STAT3 siRNAs, cells were transfected with each of the individual siRNA oligonucleotides. Three of the siRNAs silenced STAT3 and consistently induced SK1 expression, while the fourth oligonucleotide was less effective in both (Figure S11 in Additional file 2). As assessed by Akt phosphorylation, the PI3K/Akt inhibitors were effective at inhibiting their respective signalling pathways, without off-target on ERK1/2 pathway (Figure 4D-F). PI3K/Akt inhibitors wortmannin (1 μM) and LY294002 (10 μM) slightly attenuated STAT3 phosphorylation and decreased leptin-induced VEGF expression, but not SK1 expression. LY294002 has also significantly decreased the basal expression of VEGF and SK1, which could not be noticed with wortmannin (LY294002 is known bind to other targets unrelated to the PI3K family [22]).

### SKand VEGF expression is regulated by MAPK and SFKs pathways

**Figure 5:**
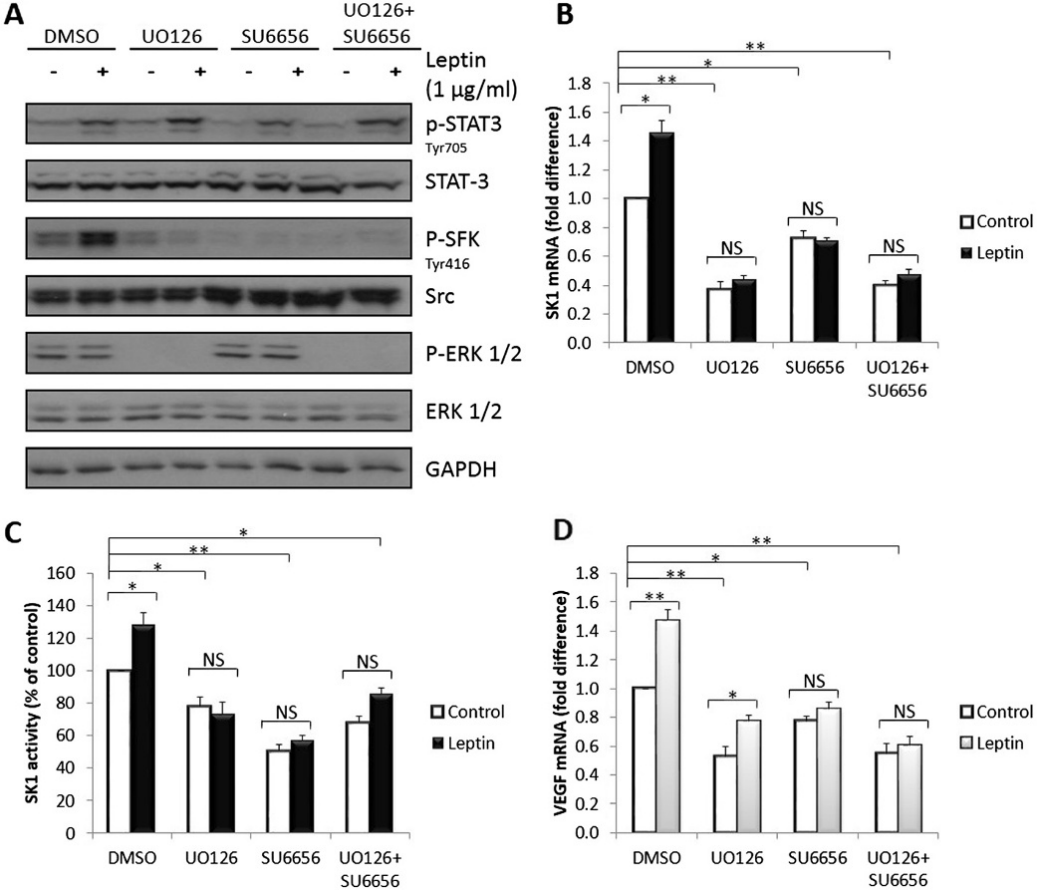
**Inhibition of ERK1/2 and SFK attenuates SK1 expression and enzymatic activity and VEGF expression.** MDAMB-231 cells were starved overnight in serum-free media and pre-treated with MEK1/2 inhibitor U0126 (10 μM) and/or SFK inhibitor SU6656 (10 μM) for 1 h followed by stimulation with 1000 ng/ml leptin for 6 h. **(A)** Cell lysates were separated on 10% SDS-PAGE gel and probed for phosphorylation of STAT3, SFK and ERK1/2. Blots are representative of three independent experiments. SK1 **(B)** and VEGF **(D)** expression and SK1 activity **(C)** were measured in cell lysates containing equal amounts of mRNA and protein. SK1 activity was measured by radiolabelling of sphingosine. For qRT-PCR, SK1 and VEGF were normalised against housekeeping genes (GAPDH, YWHAZ and UBC) and analysed using qBase software. Columns*,* mean of three independent experiments performed in triplicate; bars*,* SEM. (*, *P* <0.05; **, *P* <0.01; §, *P* <0.001; NS, not significant, *P* >0.05).

Revealing a nonessential role of JAK2 in leptin-mediated SK1 activation prompted us to identify JAK2-independent pathways. The pharmacological inhibitors were successful at inhibiting their respective targets, as assessed by the phosphorylation status of ERK and SFKs. Inhibition of MEK1/2 by UO126 and SFKs by SU6656 strongly suppressed SK1 and VEGF expression and SK1 activity (Figure 5). The combination of UO126 and SU6656 acted similarly to the stronger of the two inhibitors. While UO126 predominantly affected SK1 expression, SU6656 was most effective in inhibiting SK1 activity indicating differential, non-additive mechanisms by which these pathways affect SK1. While both inhibitors lowered basal VEGF expression, in contract to SU6656, UO126 did not inhibit leptin-induced VEGF expression. Interestingly, inhibition of ERK1/2 decreased SFKs phosphorylation, but markedly increased STAT3 phosphorylation (approximately 2.5-fold, *P* <0.001). In contrast, SFKs inhibition significantly decreased STAT3 phosphorylation, which is also noticed to a lesser extent in combination with UO126 (*P* <0.001) (Figure 5; Figure S12 in Additional file 2). Pharmacological inhibition of ERK1/2 and SFKs was verified using specific siRNA pools against ERK1/2, Src and Fyn (Figure S13-S15 in Additional file 2). Similar data was obtained in BT-549 cells, where inhibition of ERK1/2 and/or SFKs inhibited both basal and leptin-induced SK1 and VEGF expression (Figure S16 in Additional file 2).

### Knockdown of SHP2 decreases SFKs phosphorylation and SK1 expression

**Figure 6:**
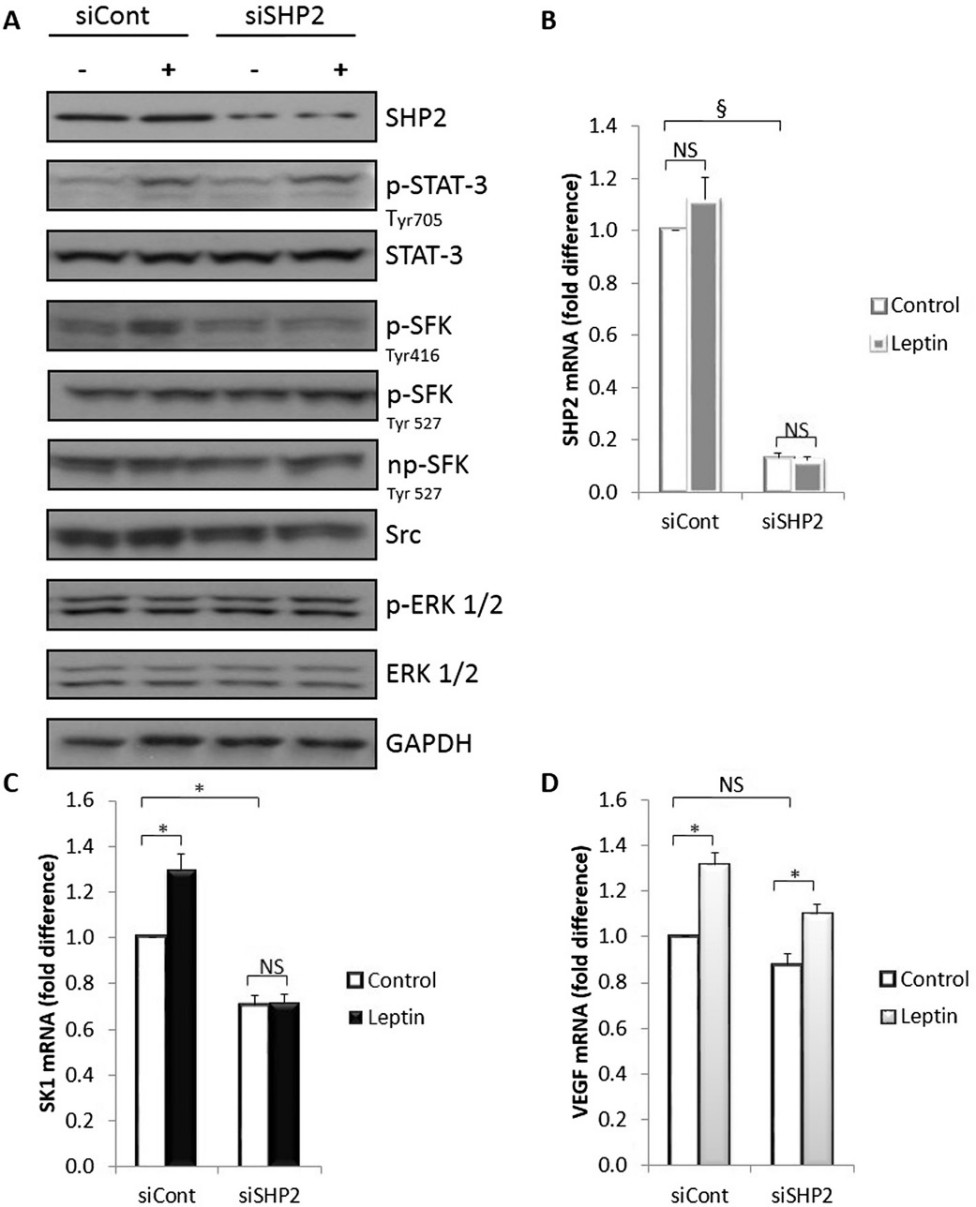
**Knockdown of SHP2 decreases SFK phosphorylation and SK1 expression.** MDAMB-231 cells were transfected with specific siRNA against SHP2 (siSHP2) or control siRNA (siCont). Then cells were starved overnight in serum-free media followed by stimulation with 1,000 ng/ml leptin for 6 h. **(A)** Cell lysates obtained were separated on a 10% SDS-PAGE gel and probed for phosphorylation of STAT3, SFK and ERK1/2. **(B)** Expression of SHP2 determined by qRT-PCR to verify knockdown efficiency. Expression of SK1 **(C)** and VEGF **(D)** mRNA determined by qRT-PCR, normalised against housekeeping genes (GAPDH, YWHAZ and UBC) and analysed using qBase software. Columns*,* mean of three independent experiments performed in triplicate; bars*,* SEM. (*, *P* <0.05; **, *P* <0.01; §, *P* <0.001; NS, not significant, *P* >0.05).

SHP2 was shown to activate SFKs, independently of its enzymatic activation, by binding to their SH3 domain [23]. Since we were interested in the pathways downstream of SHP2 which might be affected with its knockdown, the phosphorylation status of the different forms of SFKs and ERK1/2 were assessed (Figure 6). The inhibition of SHP2 expression did not lead to any change in p-SFK Tyr 527 or np-SFK Tyr 527, but reduced leptin-induced p-SFK Tyr 416 and SK1 expression by 40% and 30% respectively (Figure 6; Figure S17 in Additional file 2). Unlike Src inhibitors, SHP2 knockdown did not alter basal and leptin-mediated VEGF expression or STAT3 phosphorylation (Figure 6). Similar findings were obtained in BT-549 cells (Figure.S18 in Additional file 2).

## Discussion

Tumour microenvironment is now recognised as a new key player in cancer progression. Breast is mainly composed of adipose tissue and many studies have shown a positive association between obesity and breast cancer. Several mechanisms have been proposed to explain this link, including high levels of the adipokine leptin. Binding of leptin to its receptor LEPR-Long elicits a proliferative and angiogenic signal transduction cascade, which varies remarkably depending on cancer cell types. Similarly to leptin, an oncogenic lipid kinase SK1 was shown to be overexpressed in human breast tumours and linked with poor prognosis, yet the mechanism of its upregulation in breast cancer was not clear.

Using human clinical samples, we show for the first time that there is a strong positive correlation between LEPR-long and SK1 both in human breast tumours and metastatic LNs (Figure 1). Expression of both genes correlates between tumours and relative LNs, indicating the persistence of this pathway during cancer metastasis. A number of studies support the association between high LEPR/leptin expression and breast cancer progression [5],[6],[24], metastasis [6] and ER-negativity [24]. We show that increased expression of both LEPR and SK1 specifically correlates with metastasis in ER-negative patients and several mechanisms of SK1-driven metastasis have been suggested in other systems [25],[26]. Our data confirm previous findings showing that SK1 mRNA is expressed in both ER-positive and negative breast cancer and is increased in ER-negative tumours [17]. Both genes are strongly associated with higher BMI, which is supported by the data showing that both obesity [18] and breast cancer metastasis [27] have been correlated with higher serum levels of the SK1 product S1P. Our data is reinforced by a study showing a polymorphism at codon 109 in the leptin receptor gene, which occurs more frequently in patients who are overweight [28]. Interestingly, these patients had higher plasma leptin levels, particularly the ones with ER-negative tumour phenotype.

Importantly, we show that SK1 plays a key role in leptin-induced breast cancer cell proliferation (Figure 2), indicating a clear physiological importance of this pathway. Overexpression of SK1 in breast cancer cells was shown to promote cell growth [29], and our data show for the first time its regulation by LEPR, which functionally links obesity and breast cancer. Our data corroborates the recent finding that leptin receptor expression is required to maintain cancer stem-like properties in triple-negative breast cancer cells [30]. While it was not possible to fully differentiate the effects of SK1 knockdown on leptin signalling versus its effect on other pro-survival signalling pathways, our data give an indication that SK1 knockdown is effective counterbalancing the effects of leptin, reducing leptin-induced cell proliferation on 39% (average 72 to 120 h) vs 27% in control cells.

The mechanism of enhanced SK1 expression in ER-negative breast cancer was never elucidated. Our *in vitro* findings show a new pathway of leptin-mediated SK1 expression in ER-negative breast cancer cells where leptin induces phosphorylation of STAT3 and SFK and an increase in SK1 mRNA expression and activity (Figure 3; Figure S4-S5 in Additional file 2). Surprisingly, in ER-positive cells leptin fails to induce SFK activation or SK1 and VEGF expression despite a marked increase in STAT3 phosphorylation (Figure S6-S8 in Additional file 2), indicating the prevalence of this pathway in ER-negative cells. Indeed, MDAMB-231 cells form more aggressive tumours than MCF-7 cells [31] and secrete higher levels of leptin and VEGF [12]. The differential activation of leptin-mediated signalling pathways across different breast cancer cell lines may further account for the distinctive activation profile of SK1. In contrast, to leptin signalling, the clear role of SK1 in response to ER-α was described previously in detail [32]. Therefore, further studies are required to investigate the differential response of SK1 to leptin depending on ER expression profiles.

SFKs play an important role in breast cancer oncogenesis. Importantly, to our knowledge, leptin-mediated activation of SFKs has not been previously reported in breast cancer and here we provide the first evidence of leptin-mediated SFKs phosphorylation at Tyr 416 in ER-negative breast cancer cell lines (Figure 3). Previous studies in other cell systems indicate that SFKs can induce VEGF expression [33], and increase SK1 expression and enzymatic activity [34]. Importantly, recent evidence suggests that in breast cancer high nuclear localisation of SK1, combined with high levels of cytoplasmic p-SFK (Tyr 416) or Lyn shortens disease recurrence time [35]. However, it is not clear what caused the high level of SFK phosphorylation observed in the absence of leptin at 1 and 24 h (Figure 3A). Taken into consideration the pattern of activation observed with regards to time and the treatment, it is possible that the initial SFK phosphorylation induced by leptin at 10 min and 6 h was followed by a desensitization phase, which has led to relative higher levels in control in comparison with leptin-stimulated cells. The activation at 24 h could be also explained by a general desensitisation to the signal as leptin signalling wears off and as metastatic cancer cells produce their own growth/proliferation factors.

**Figure 7:**
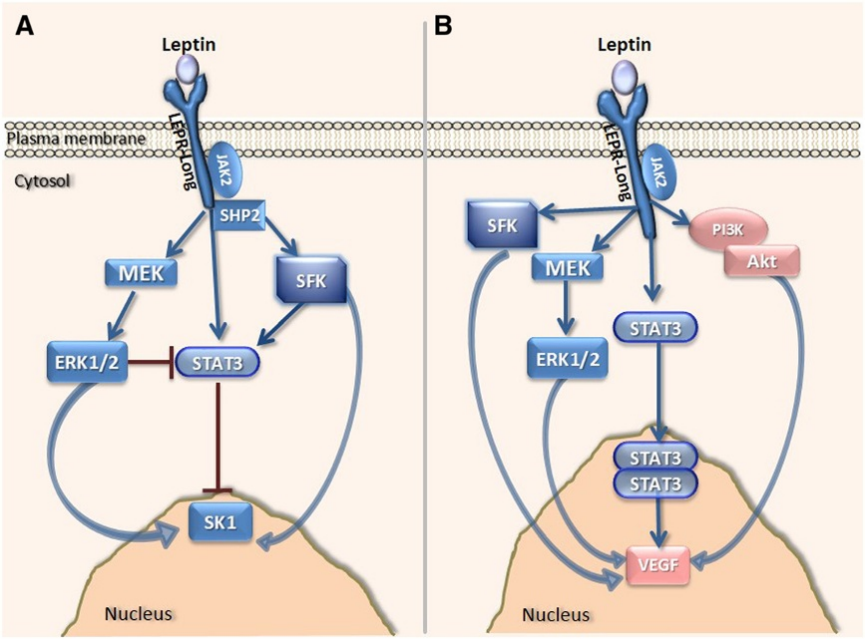
**Overall mechanism of leptin-mediated activation of downstream signalling. (A)** Leptin activates SK1 through ERK1/2 and SHP2/SFKs-mediated mechanisms. ERK1/2 inhibits STAT3 activation, and STAT3 inhibits SK1 expression. **(B)** Activation of VEGF expression occurs via JAK2/STAT3, PI3K/Akt, SFKs and ERK1/2-mediated mechanisms.

Unexpectedly, JAK2 silencing did not alter leptin-induced STAT3 phosphorylation, and SK1 expression, while, similarly to a previous report [36], knockdown of either JAK2 or STAT3 abolished leptin-induced VEGF expression (Figure 4; Figure S9-S11 in Additional file 2). This suggests the presence of two distinct leptin-driven pathways [10] that regulate the expression of these genes in ER-negative breast cancer cells. Indeed, here we provide the first evidence that in ER-negative breast cancer cell lines ERK1/2 and SFKs pathways regulate leptin-mediated increase in SK1 expression and activity (Figures 5, 7, Figure S12-S16 in Additional file 2), while JAK2/STAT3, PI3K/Akt, ERK1/2 and SFKs pathways regulate expression of VEGF (Figures 4, 5, 7).

Differential inhibition of SK1 activity and expression by UO126 and SU6656 shows that SFKs mainly affect the enzyme activity whereas ERK1/2 affect mostly its basal transcriptional levels. Our data are consistent with a previous report showing that UO126 decreases PMA-induced SK1 expression [37]. Concurrent inhibition of ERK1/2 and SFKs signalling did not provide any synergistic or additive effect, but displayed a phenotype consistent with the stronger inhibitor; UO126. It is therefore highly probable that these molecules are involved in separate signalling pathways; nonetheless, they coordinate leptin responses through a similar downstream mechanism involving SK1.

An unexpected observation is the reduction of SFK phosphorylation after treatment with UO126. A similar reduction has been shown in response to UO126 in rat intestinal epithelial cells [38] and a similar reduction in p-SFK was observed when using siERK1/2 (Figure S13 in Additional file 2), ruling out the non-specific inhibitor effects. ERK1/2 was reported to inhibit the ubiquitination pathway [39], which is known to contribute to p-SFKs degradation in the absence of downstream signalling [40],[41]. In all of our experiments, the total SFK levels remained unchanged and it was only p-SFK that was downregulated. We therefore conclude that it is possible that: (a) inhibition of ERK may lead to activation of the ubuiqitination pathway and subsequent degradation of p-SFK or (b) there is an unknown feedback mechanism linking ERK1/2 and SFK phosphorylation.

One interesting finding is that MEK1/2 inhibition or knockdown increases STAT3 phosphorylation (Figure 5; Figure S13, S16 in Additional file 2). It was shown that mutation in the LEPR site responsible for ERK activation leads to enhanced STAT3 signalling [42], while expression of constitutively active MEK inhibits STAT3 activation [43]. These data suggest that ERKs and STAT3 form a negative feedback loop, limiting the intensity of STAT3 activation [43].

SHP2 has an oncogenic role in triple-negative breast cancer [44], and functions as an adaptor protein in LEPR signalling to ERK1/2 [42]. SHP2 was also shown to induce Src activation through enzymatic [45] and non-enzymatic mechanisms [23]. Our data show for the first time that SHP2 knockdown reduces p-SFK Tyr 416, but not Tyr 527 in leptin-treated MDA-MB-231 and BT-549 cells and is critical for leptin-induced SK1 expression (Figure 6; Figure S17, S18 in Additional file 2).

Breast cancer clinical phenotype is a key factor in designing therapeutic approaches. In ER-positive tumours SK1 expression was reported to have no detrimental effect [46], on the contrary, high SK1 expression in ER-negative tumours is associated with shorter disease-specific survival [17],[47]. Therefore, SK1 inhibitors might be of little use for the treatment of ER-positive breast cancer [48], while our data indicate that they may have some potential in treatment of ER-negative tumours, specifically in the context of high BMI. In other cancer systems we have previously demonstrated a significant potential for the SK1-targeting therapies, specifically in combination with docetaxel chemotherapy [19],[49]-[51]. In triple-negative breast cancer models inhibition of SK1 decreases cell proliferation [52] and reduces primary tumour size and LN metastasis [27].

## Conclusions

Our study identifies SK1 as a new player in leptin-induced response in ER-negative breast cancer cells and tissues. Contrary to well-studied leptin-induced VEGF expression, the findings in this study provide a novel SHP2/SFKs- and ERK1/2-dependent mechanism of leptin-mediated SK1 regulation. Recent clinical data links obesity with ER- and PR-negative tumours and poor overall survival in patients with breast cancer [53]. A recent WINS trial demonstrated that low-fat diet and corresponding weight loss showed a relapse-free survival benefit only in ER-negative breast cancer patients [54]. While this effect is clearly multifactorial and may be linked to changes in sex hormone levels, insulin, adipokines and inflammatory response, leptin reduction may play a role in the achieved outcome and our data show a new mechanism of leptin-mediated effect on breast cancer signalling (Figure 7). The findings in this work point to the possibility of targeting SK1 in ER-negative tumours and obese individuals to deter breast cancer progression. Further investigation is required to delineate the exact mechanism of SK1 expression as well as its subsequent influence on breast tumour progression.

## Authors' contributions

HA performed the majority of experiments, analysed and interpreted the data, and drafted the manuscript. JK performed clinical experiments, analysed data and helped drafting the manuscript. AF performed clinical experiments, analysed data and helped drafting the manuscript. JW performed data analysis and critically revised the manuscript. OB participated in the design of the study and performed the statistical analysis of clinical data. AZ participated in the design of the study and performed the statistical analysis of clinical data. MW performed data analysis, worked with clinical samples and critically revised the manuscript. JS performed data analysis and critically revised the manuscript. EY supervised experiments, performed data analysis and critically revised the manuscript. DP generated the idea, designed, coordinated and supervised all work, drafted and revised the manuscript. All authors have read and approved the final manuscript.

## Additional files

## Electronic supplementary material

Additional file 1: Table S1.: Patient characteristics with clinicopathological parameters of breast cancer patients. **Table S2.** Primer sequences used. Table S3. Sequences of siRNA oligonucleotides used. **Table S4.** Parameters defining the best multivariate linear regression model for both genes and the corresponding coefficients. **Table S5.** Odds ratios for the pair of parameters, which mostly influence the outcome for LEPR. **Table S6.** Odds ratios for the pair of parameters, which mostly influence the outcome for SPHK1. (DOCX 27 KB)

Additional file 2: Figure S1.: LEPR-Long and SK1 expression is slightly elevated in triple-negative breast cancer patients. **Figure S2.** Leptin does not increase the proliferation of BT-549 cells in the absence of SK1 signalling. **Figure S3.** Leptin activates p-STAT3 and P-SFK in MDAMB-231 cells. **Figure S4.** Leptin activates p-STAT3 and P-SFK in BT-549 cells. **Figure S5.** Leptin increases SK1 expression and enzymatic activity and VEGF expression in BT-549 cells. **Figure S6.** Leptin activates p-STAT3 in a dose-dependent manner In MCF-7. **Figure S7.** Leptin does not increase SK1 expression and enzymatic activity and VEGF expression in MCF-7 cells. **Figure S8.** Leptin activates p-STAT3 in BT-474 cells. **Figure S9.** JAK2 silencing or PI3K/Akt inhibition does not abrogate STAT3 phosphorylation. **Figure S10.** STAT3 silencing potentiates SK1 expression. **Figure S11.** STAT3 siRNA silenced STAT3 and induced SK1 expression. **Figure S12.** Inhibition of ERK1/2 and SFK modulates STAT3 phosphorylation. **Figure S13.** ERK silencing increases STAT3 phosphorylation. **Figure S14.** ERK silencing attenuates SK1 expression and enzymatic activity and VEGF expression. **Figure S15.** SFK phosphorylation at site 416 is important for leptin signalling. **Figure S16.** Inhibition of ERK1/2 and SFK modulates STAT3 phosphorylation and attenuates SK1 and VEGF expression. **Figure S17.** Knockdown of SHP2 decreases SFK phosphorylation. **Figure S18.** Knockdown of SHP2 decreases SFK phosphorylation and SPHK1 expression. (PDF 2 MB)

Below are the links to the authors’ original submitted files for images.

Authors’ original file for figure 1

Authors’ original file for figure 2

Authors’ original file for figure 3

Authors’ original file for figure 4

Authors’ original file for figure 5

Authors’ original file for figure 6

Authors’ original file for figure 7

## Abbreviations

18S: eukaryotic 18S rRNA
BMI: body mass index
DMEM: Dulbecco’s modified Eagle’s medium
ER: oestrogen receptor
ERK1/2: extracellular signal-regulated kinases 1/2
FCS: foetal calf serum
FFPE: formalin-fixed and paraffin-embedded
GAPDH: glyceraldehyde-3-phosphate dehydrogenase
GUSB: beta glucuronidase
JAK2: janus kinase 2
LEPR: leptin receptor
LEPR-Long: longest isoform of LEPR
LN: lymph node
MRPL19: mitochondrial ribosomal protein L19
MTT: 3-(4,5-dimethylthiazol-2-yl)-2,5-diphenyl tetrazolium bromide
OR: odds ratio
PI3K: phosphatidylinositol 3-kinase
PBS: phosphate-buffered saline
PR: progesterone receptors
ROX: 6-carboxyl-X-rhodamine
S1P: sphingosine-1-phosphate
SEM: standard error of the mean
SFKs: Src family kinases
SHP2: Src-homology 2 domain-containing phosphatase 2
siRNA: small interfering RNA
SK1: sphingosine kinase 1
STAT3: signal transducer and activator of transcription 3
TBP: TATA box binding protein
UBC: ubiquitin C
VEGF: vascular endothelial growth factor
YWAZH: tyrosine-3-monooxy-genase/tryptophan 5-mono-oxygenase activation protein

## References

[1] Kelly T, Yang W, Chen CS, Reynolds K, He J (2008). Global burden of obesity in 2005 and projections to 2030. Int J Obes.

[2] Cleary MP, Grossmann ME (2009). Obesity and breast cancer: the estrogen connection. Endocrinology.

[3] Maccio A, Madeddu C, Gramignano G, Mulas C, Floris C, Massa D, Astara G, Chessa P, Mantovani G (2010). Correlation of body mass index and leptin with tumor size and stage of disease in hormone-dependent postmenopausal breast cancer: preliminary results and therapeutic implications. J Mol Med (Berl).

[4] Anderson GL, Neuhouser ML (2012). Obesity and the risk for premenopausal and postmenopausal breast cancer. Cancer Prev Res.

[5] Miyoshi Y, Funahashi T, Tanaka S, Taguchi T, Tamaki Y, Shimomura I, Noguchi S (2006). High expression of leptin receptor mRNA in breast cancer tissue predicts poor prognosis for patients with high, but not low, serum leptin levels. Int J Cancer.

[6] Ishikawa M, Kitayama J, Nagawa H (2004). Enhanced expression of leptin and leptin receptor (OB-R) in human breast cancer. Clin Cancer Res.

[7] Zheng Q, Dunlap SM, Zhu J, Downs-Kelly E, Rich JN, Hursting SD, Berger NA, Reizes O (2011). Leptin deficiency suppresses MMTV-Wnt-1 mammary tumor growth and abrogates tumor initiating cell survival. Endocr Relat Cancer.

[8] Zabeau L, Lavens D, Peelman F, Eyckerman S, Vandekerckhove J, Tavernier J (2003). The ins and outs of leptin receptor activation. FEBS Lett.

[9] Ando S, Catalano S (2011). The multifactorial role of leptin in driving the breast cancer microenvironment. Nat Rev Endocrinol.

[10] Jiang L, Li Z, Rui L (2008). Leptin stimulates both JAK2-dependent and JAK2-independent signaling pathways. J Biol Chem.

[11] Ray A, Nkhata KJ, Cleary MP (2007). Effects of leptin on human breast cancer cell lines in relationship to estrogen receptor and HER2 status. Int J Oncol.

[12] Gonzalez RR, Watters A, Xu YB, Singh UP, Mann DR, Rueda BR, Penichet ML (2009). Leptin-signaling inhibition results in efficient anti-tumor activity in estrogen receptor positive or negative breast cancer. Breast Cancer Res.

[13] Alshaker H, Sauer L, Monteil D, Ottaviani S, Srivats S, Bohler T, Pchejetski D (2013). Therapeutic potential of targeting SK1 in human cancers. Adv Cancer Res.

[14] French KJ, Schrecengost RS, Lee BD, Zhuang Y, Smith SN, Eberly JL, Yun JK, Smith CD (2003). Discovery and evaluation of inhibitors of human sphingosine kinase. Cancer Res.

[15] Van Brocklyn JR, Jackson CA, Pearl DK, Kotur MS, Snyder PJ, Prior TW (2005). Sphingosine kinase-1 expression correlates with poor survival of patients with glioblastoma multiforme: roles of sphingosine kinase isoforms in growth of glioblastoma cell lines. J Neuropathol Exp Neurol.

[16] Ling BB, Chen LF, Alcorn J, Ma BH, Yang JA (2011). Sphingosine-1-phosphate: a potential therapeutic agent against human breast cancer. Invest New Drugs.

[17] Ruckhaberle E, Rody A, Engels K, Gaetje R, von Minckwitz G, Schiffmann S, Grosch S, Geisslinger G, Holtrich U, Karn T, Kaufmann M (2008). Microarray analysis of altered sphingolipid metabolism reveals prognostic significance of sphingosine kinase 1 in breast cancer. Breast Cancer Res Treat.

[18] Blachnio-Zabielska AU, Pulka M, Baranowski M, Nikolajuk A, Zabielski P, Gorska M, Gorski J (2012). Ceramide metabolism is affected by obesity and diabetes in human adipose tissue. J Cell Physiol.

[19] Sauer L, Nunes J, Salunkhe V, Skalska L, Kohama T, Cuvillier O, Waxman J, Pchejetski D (2009). Sphingosine kinase 1 inhibition sensitizes hormone-resistant prostate cancer to docetaxel. Int J Cancer.

[20] Pchejetski D, Nunes J, Coughlan K, Lall H, Pitson SM, Waxman J, Sumbayev VV (2011). The involvement of sphingosine kinase 1 in LPS-induced Toll-like receptor 4-mediated accumulation of HIF-1alpha protein, activation of ASK1 and production of the pro-inflammatory cytokine IL-6. Immunol Cell Biol.

[21] Bonhoure E, Pchejetski D, Aouali N, Morjani H, Levade T, Kohama T, Cuvillier O (2006). Overcoming MDR-associated chemoresistance in HL-60 acute myeloid leukemia cells by targeting sphingosine kinase-1. Leukemia.

[22] Gharbi SI, Zvelebil MJ, Shuttleworth SJ, Hancox T, Saghir N, Timms JF, Waterfield MD (2007). Exploring the specificity of the PI3K family inhibitor LY294002. Biochem J.

[23] Walter AO, Peng ZY, Cartwright CA (1999). The Shp-2 tyrosine phosphatase activates the Src tyrosine kinase by a non-enzymatic mechanism. Oncogene.

[24] Goodwin PJ, Ennis M, Fantus IG, Pritchard KI, Trudeau ME, Koo J, Hood N (2005). Is leptin a mediator of adverse prognostic effects of obesity in breast cancer?. J Clin Oncol.

[25] Maceyka M, Alvarez SE, Milstien S, Spiegel S (2008). Filamin A links sphingosine kinase 1 and sphingosine-1-phosphate receptor 1 at lamellipodia to orchestrate cell migration. Mol Cell Biol.

[26] Bryan L, Paugh BS, Kapitonov D, Wilczynska KM, Alvarez SM, Singh SK, Milstien S, Spiegel S, Kordula T (2008). Sphingosine-1-phosphate and interleukin-1 independently regulate plasminogen activator inhibitor-1 and urokinase-type plasminogen activator receptor expression in glioblastoma cells: implications for invasiveness. Mol Cancer Res.

[27] Nagahashi M, Ramachandran S, Kim EY, Allegood JC, Rashid OM, Yamada A, Zhao R, Milstien S, Zhou H, Spiegel S, Takabe K (2012). Sphingosine-1-phosphate produced by sphingosine kinase 1 promotes breast cancer progression by stimulating angiogenesis and lymphangiogenesis. Cancer Res.

[28] Liu CL, Chang YC, Cheng SP, Chern SR, Yang TL, Lee JJ, Guo IC, Chen CP (2007). The roles of serum leptin concentration and polymorphism in leptin receptor gene at codon 109 in breast cancer. Oncology.

[29] Nava VE, Hobson JP, Murthy S, Milstien S, Spiegel S (2002). Sphingosine kinase type 1 promotes estrogen-dependent tumorigenesis of breast cancer MCF-7 cells. Exp Cell Res.

[30] Zheng Q, Banaszak L, Fracci S, Basali D, Dunlap SM, Hursting SD, Rich JN, Hjlemeland AB, Vasanji A, Berger NA, Lathia JD, Reizes O (2013). Leptin receptor maintains cancer stem-like properties in triple negative breast cancer cells. Endocr Relat Cancer.

[31] Ray A, Nkhata KJ, Grande JP, Cleary MP (2007). Diet-induced obesity and mammary tumor development in relation to estrogen receptor status. Cancer Lett.

[32] Sukocheva O, Wadham C, Holmes A, Albanese N, Verrier E, Feng F, Bernal A, Derian CK, Ullrich A, Vadas MA, Xia P (2006). Estrogen transactivates EGFR via the sphingosine 1-phosphate receptor Edg-3: the role of sphingosine kinase-1. J Cell Biol.

[33] Mukhopadhyay D, Tsiokas L, Zhou XM, Foster D, Brugge JS, Sukhatme VP (1995). Hypoxic induction of human vascular endothelial growth factor expression through c-Src activation. Nature.

[34] Sobue S, Murakami M, Banno Y, Ito H, Kimura A, Gao S, Furuhata A, Takagi A, Kojima T, Suzuki M, Nozawa Y, Murate T (2008). v-Src oncogene product increases sphingosine kinase 1 expression through mRNA stabilization: alteration of AU-rich element-binding proteins. Oncogene.

[35] Ohotski J, Edwards J, Elsberger B, Watson C, Orange C, Mallon E, Pyne S, Pyne NJ (2013). Identification of novel functional and spatial associations between sphingosine kinase 1, sphingosine 1-phosphate receptors and other signaling proteins that affect prognostic outcome in estrogen receptor-positive breast cancer. Int J Cancer.

[36] Gonzalez-Perez RR, Xu Y, Guo S, Watters A, Zhou W, Leibovich SJ (2010). Leptin upregulates VEGF in breast cancer via canonic and non-canonical signalling pathways and NFkappaB/HIF-1alpha activation. Cell Signal.

[37] Nakade Y, Banno Y, T-Koizumi K, Hagiwara K, Sobue S, Koda M, Suzuki M, Kojima T, Takagi A, Asano H, Nozawa Y, Murate T (2003). Regulation of sphingosine kinase 1 gene expression by protein kinase C in a human leukemia cell line, MEG-O1. Biochim Biophys Acta.

[38] Kang ES, Oh MA, Lee SA, Kim TY, Kim SH, Gotoh N, Kim YN, Lee JW (2007). EGFR phosphorylation-dependent formation of cell-cell contacts by Ras/Erks cascade inhibition. Biochim Biophys Acta.

[39] Miao J, Xiao Z, Kanamaluru D, Min G, Yau PM, Veenstra TD, Ellis E, Strom S, Suino-Powell K, Xu HE, Kemper JK (2009). Bile acid signaling pathways increase stability of Small Heterodimer Partner (SHP) by inhibiting ubiquitin-proteasomal degradation. Genes Dev.

[40] Hakak Y, Martin GS (1999). Ubiquitin-dependent degradation of active Src. Curr Biol.

[41] Lu Z, Hunter T (2009). Degradation of activated protein kinases by ubiquitination. Annu Rev Biochem.

[42] Bjorbaek C, Buchholz RM, Davis SM, Bates SH, Pierroz DD, Gu H, Neel BG, Myers MG, Flier JS (2001). Divergent roles of SHP-2 in ERK activation by leptin receptors. J Biol Chem.

[43] Sengupta TK, Talbot ES, Scherle PA, Ivashkiv LB (1998). Rapid inhibition of interleukin-6 signaling and Stat3 activation mediated by mitogen-activated protein kinases. Proc Natl Acad Sci U S A.

[44] Aceto N, Sausgruber N, Brinkhaus H, Gaidatzis D, Martiny-Baron G, Mazzarol G, Confalonieri S, Quarto M, Hu G, Balwierz PJ, Pachkov M, Elledge SJ, van Nimwegen E, Stadler MB, Bentires-Alj M (2012). Tyrosine phosphatase SHP2 promotes breast cancer progression and maintains tumor-initiating cells via activation of key transcription factors and a positive feedback signaling loop. Nat Med.

[45] Zhang SQ, Yang W, Kontaridis MI, Bivona TG, Wen G, Araki T, Luo J, Thompson JA, Schraven BL, Philips MR, Neel BG (2004). Shp2 regulates SRC family kinase activity and Ras/Erk activation by controlling Csk recruitment. Mol Cell.

[46] Long JS, Edwards J, Watson C, Tovey S, Mair KM, Schiff R, Natarajan V, Pyne NJ, Pyne S (2010). Sphingosine kinase 1 induces tolerance to human epidermal growth factor receptor 2 and prevents formation of a migratory phenotype in response to sphingosine 1-phosphate in estrogen receptor-positive breast cancer cells. Mol Cell Biol.

[47] Ohotski J, Long JS, Orange C, Elsberger B, Mallon E, Doughty J, Pyne S, Pyne NJ, Edwards J (2012). Expression of sphingosine 1-phosphate receptor 4 and sphingosine kinase 1 is associated with outcome in oestrogen receptor-negative breast cancer. Br J Cancer.

[48] Pyne NJ, Tonelli F, Lim KG, Long J, Edwards J, Pyne S (2012). Targeting sphingosine kinase 1 in cancer. Adv Biol Regul.

[49] Pchejetski D, Bohler T, Brizuela L, Sauer L, Doumerc N, Golzio M, Salunkhe V, Teissie J, Malavaud B, Waxman J, Cuvillier O (2010). FTY720 (fingolimod) sensitizes prostate cancer cells to radiotherapy by inhibition of sphingosine kinase-1. Cancer Res.

[50] Pchejetski D, Doumerc N, Golzio M, Naymark M, Teissie J, Kohama T, Waxman J, Malavaud B, Cuvillier O (2008). Chemosensitizing effects of sphingosine kinase-1 inhibition in prostate cancer cell and animal models. Mol Cancer Ther.

[51] Pchejetski D, Golzio M, Bonhoure E, Calvet C, Doumerc N, Garcia V, Mazerolles C, Rischmann P, Teissie J, Malavaud B, Cuvillier O (2005). Sphingosine kinase-1 as a chemotherapy sensor in prostate adenocarcinoma cell and mouse models. Cancer Res.

[52] Antoon JW, White MD, Driver JL, Burow ME, Beckman BS (2012). Sphingosine kinase isoforms as a therapeutic target in endocrine therapy resistant luminal and basal-A breast cancer. Exp Biol Med.

[53] Turkoz FP, Solak M, Petekkaya I, Keskin O, Kertmen N, Sarici F, Arik Z, Babacan T, Ozisik Y, Altundag K (2013). The prognostic impact of obesity on molecular subtypes of breast cancer in premenopausal women. J BUON.

[54] Chlebowski RT, Blackburn GL, Thomson CA, Nixon DW, Shapiro A, Hoy MK, Goodman MT, Giuliano AE, Karanja N, McAndrew P, Hudis C, Butler J, Merkel D, Kristal A, Caan B, Michaelson R, Vinciguerra V, Del Prete S, Winkler M, Hall R, Simon M, Winters BL, Elashoff RM (2006). Dietary fat reduction and breast cancer outcome: interim efficacy results from the Women’s Intervention Nutrition Study. J Natl Cancer Inst.

