# Supplementary material for: Leptin induces upregulation of sphingosine kinase 1 in oestrogen receptor-negative breast cancer via Src family kinase-mediated, janus kinase 2-independent pathway
Source: Breast Cancer Res. 2014 Oct 25;16:426. doi: 10.1186/s13058-014-0426-6 (PMC4303110; doi:10.1186/s13058-014-0426-6)
Supplement: Supplementary file 1 — Additional file 1: Table S1.: Patient characteristics with clinicopathological parameters of breast cancer patients. Table S2. Primer sequences used. Table S3. Sequences of siRNA oligonucleotides used. Table S4. Parameters defining the best multivariate linear regression model for both genes and the corresponding coefficients. Table S5. Odds ratios for the pair of parameters, which mostly influence the outcome for LEPR. Table S6. Odds ratios for the pair of parameters, which mostly influence the outcome for SPHK1. (DOCX 27 KB) [file 13058_2014_426_MOESM1_ESM.docx]

**Supplementary tables**

# Table S1. Patient characteristics with clinicopathological parameters of breast cancer patients

| **Characteristics** | **Patients (n[%])** |
| --- | --- |
| **Age (Years)** |  |
| **30-39** | 12 [17.4] |
| **40-49** | 17 [24.6] |
| **50-59** | 15 [21.7] |
| **>60** | 25 [36.2] |
| **BMI** |  |
| **18.6-24.9** | 27 [39.1] |
| **25-29.9** | 18 [26.1] |
| **>30** | 18 [26.1] |
| **Unknown** | 6 [8.7] |
| **Menopausal status** |  |
| **pre** | 29 [42.0] |
| **post** | 39 [56.5] |
| **Unknown** | 1 [1.4] |
| **Nodal status** |  |
| **1-3** | 46 [66.7] |
| **≥4** | 23 [33.3] |
| **Tumour size (mm)** |  |
| **0-20** | 32 [46.4] |
| **21-50** | 28 [40.6] |
| **>51** | 6 [8.7] |
| **Unknown** | 3 [4.3] |
| **Tumour grade** |  |
| **I** | 4 [5.8] |
| **II** | 30 [43.5] |
| **III** | 35 [50.7] |
| **Oestrogen receptor status** |  |
| **Positive** | 35 [50.7] |
| **Negative** | 34 [49.3] |
| **Progesterone receptor status** |  |
| **Positive** | 35 [50.7] |
| **Negative** | 34 [49.3] |
| **Her2 status** |  |
| **Positive** | 23 [33.3] |
| **Negative** | 46 [66.7] |
| **EGFR overexpression** |  |
| **Positive** | 10 [14.5] |
| **Negative** | 21 [30.4] |
| **Unknown** | 38 [55.1] |
| **Histologic type** |  |
| **Invasive ductal carcinoma** | 67 [97.1] |
| **Invasive lobular carcinoma** | 2 [2.9] |

**Table S2. Primer sequences used**

| **Symbol** | **Gene Name** | **Primer sequence** | |
| --- | --- | --- | --- |
| **SK1** | Sphingosine kinase 1 | Fw: TATGAATGCCCCTACTTGGTATTG  Rv: GCCTCGCTAACCATCAATTCC | |
| **SHP2 (PTPN11)** | Protein tyrosine phosphatase,  non-receptor type 11 | Fw: GCTGCCATTCTCACTGACAA Rv: CACCTGTGTTCTGAAGCAATTC | |
| **VEGF** | Vascular endothelial growth factor | Fw: TCGAGTACATCTTCAAGCC Rv: TGATCCGCATAATCTGCAT | |
| **LEPR-Long** | Leptin receptor (for longest version, transcript variant 1) | Fw: GGGGTCACCTCAATCAAAAAGA Rv: AGCAACTGTCCTGGAGAACT | |
| **LEPR-Total** | Leptin receptor (Common domain of all isoforms) | Fw: ATGCCACCAAATTCAACCTATG Rv: GCTGTCTCATAATGTCCATTCG | |
|  |  | **Assay ID** |  |
| **GUSB** | glucuronidase, beta | Hs99999908_m1 |  |
| **TBP** | TATA box binding protein | Hs00427620_m1 |  |
| **18S** | eukaryotic 18S rRNA | Hs03003631_g1 |  |
| **MRPL19** | mitochondrial ribosomal protein L19 | Hs00608519_m1 |  |

**Table S3. Sequences of siRNA oligonucleotides used**

| **Target** | **Gene ID** | **Target sequence** | **Catalogue number** |
| --- | --- | --- | --- |
| Src | 6714 | GAGAACCUGGUGUGCAAAG | D-003175-05 |
|  |  | CGUCCAAGCCGCAGACUCA | D-003175-06 |
|  |  | CCUCAGGCAUGGCGUACGU | D-003175-07 |
|  |  | CCAAGGGCCUCAACGUGAA | D-003175-12 |
| FYN | 2534 | GCUCUGAAAUUACCAAAUC | D-003140-09 |
|  |  | CGCAUGAAUUAUAUCCAUA | D-003140-10 |
|  |  | CUGUGAAGCAUUCGAGACA | D-003140-23 |
|  |  | CAGGAAUGGCUUACAACGA | D-003140-24 |
| JAK2 | 3717 | GAGCAAAGAUCCAAGACUA | D-003146-06 |
|  |  | GCCAGAAACUUGAAACUUA | D-003146-07 |
|  |  | GAUCCUGGCAUUAGUAUUA | D-003146-09 |
|  |  | ACAGAAUGCUGGAACAAUA | D-003146-10 |
| STAT3 | 6774 | GGAGAAGCAUCGUGAGUGA | D-003544-02 |
|  |  | CCACUUUGGUGUUUCAUAA | D-003544-03 |
|  |  | UCAGGUUGCUGGUCAAAUU | D-003544-04 |
|  |  | CGUUAUAUAGGAACCGUAA | D-003544-19 |
| ERK1 (MAPK3) | 5595 | GACCGGAUGUUAACCUUUA | J-003592-07 |
|  |  | CCUGCGACCUUAAGAUUUG | J-003592-08 |
|  |  | CCAAUAAACGGAUCACAGU | J-003592-09 |
|  |  | AGACUGACCUGUACAAGUU | J-003592-10 |
| ERK2 (MAPK1) | 5594 | ACACCAACCUCUCGUACAU | J-003555-14 |
|  |  | GGUGUGCUCUGCUUAUGAU | J-003555-13 |
|  |  | CACCAACCAUCGAGCAAAU | J-003555-12 |
|  |  | UCGAGUAGCUAUCAAGAAA | J-003555-11 |
| SHP2 (PTPN11) | 5781 | GAACAUCACGGGCAAUUAA | J-003947-09 |
|  |  | GAAGCACAGUACCGAUUUA | J-003947-10 |
|  |  | GGAGAUGGUUUCACCCAAA | J-003947-11 |
|  |  | GGACGUUCAUUGUGAUUGA | J-003947-12 |
| SK1 | UCACGCUGAUGCUCACUGA(TT) | | |
|  | UCAGUGAGCAUCAGCGUGA(AG) | | |
| Negative control | UCUACUCCUUCUGCAACCC(TT) | | |
|  | GGGUUGCAGAAGGAGUAGA(TT) | | |

**Table S4. Parameters defining the best multivariate linear regression model for both genes and the corresponding coefficients**

| **LN LEPR - Tumour LEPR** | | | **LN SPHK1 - Tumour SPHK1** | | |
| --- | --- | --- | --- | --- | --- |
| **Variable** | **Coefficient** | **p-value** | **Variable** | **Coefficient** | **p-value** |
| **Intercept** | 1.299 | <0.001 | **Intercept** | 1.606 | <0.001 |
| **ER+ vs ER-** | -0.882 | 0.019 | **ER+ vs ER-** | -1.693 | 0.003 |
| **Tum Size <20mm vs 20-50mm** | -0.463 | 0.223 | **Tum Size <20mm vs 20-50mm** | -1.246 | 0.034 |
| **Tum Size <20mm vs >50mm** | -1.884 | 0.012 | **Tum Size <20mm vs >50mm** | -2.155 | 0.008 |

To find the parameters included in the best linear regression model two outcomes were considered: (LN LEPR - Tumour LEPR) and (LN SPHK1 - Tumour SPHK1), and the best model has been found via stepwise linear regression based on the minimization of Akaike information criterion. The clinical parameters have been considered as categorical data.

**Table S5. ORs for the pair of parameters, which mostly influence the outcome for LEPR**

| **Tumour Grade** | **HER2 Status** | **No of samples** | **OR** | **CI** | **p-value** |
| --- | --- | --- | --- | --- | --- |
| I+II | - | 24 | 1 |  |  |
| I+II | + | 10 | 9.818 | 1.485-196.595 | 0.044 |
| III | - | 22 | 4.091 | 1.097-17.927 | 0.044 |
| III | + | 13 | 1.273 | 0.324-5.119 | 0.729 |

The Odds Ratio (OR) for all pairs of parameters for considered outcome (LN LEPR - Tumour LEPR) has been calculated with respect to tertiles of tumour grade and HER2 status. The results for pairs with most significant ORs (calculated via logistic regression) are presented.

**Table S6. ORs for the pair of parameters, which mostly influence the outcome for SPHK1**

| **ER status** | **BMI** | **No of samples** | **OR** | **CI** | **p-value** |
| --- | --- | --- | --- | --- | --- |
| ER- | <25 | 16 | 1 |  |  |
| ER- | >25 | 15 | 9.9 | 0.012-0.564 | 0.016 |
| ER+ | <25 | 11 | 9.625 | 0.012-0.634 | 0.022 |
| ER+ | >25 | 21 | 6.111 | 0.021-0.823 | 0.043 |

The Odds Ratio (OR) for all pairs of parameters has been calculated with respect to the first tertiles of tumour grade and BMI status. The results for pairs with most significant ORs (calculated via logistic regression) are presented. Here the considered outcome was (Tumour SPHK1 - LN SPHK1).
