# Supplementary material for: Leptin induces upregulation of sphingosine kinase 1 in oestrogen receptor-negative breast cancer via Src family kinase-mediated, janus kinase 2-independent pathway
Source: Breast Cancer Res. 2014 Oct 25;16:426. doi: 10.1186/s13058-014-0426-6 (PMC4303110; doi:10.1186/s13058-014-0426-6)
Supplement: Supplementary file 2 — Additional file 2: Figure S1.: LEPR-Long and SK1 expression is slightly elevated in triple-negative breast cancer patients. Figure S2. Leptin does not increase the proliferation of BT-549 cells in the absence of SK1 signalling. Figure S3. Leptin activates p-STAT3 and P-SFK in MDAMB-231 cells. Figure S4. Leptin activates p-STAT3 and P-SFK in BT-549 cells. Figure S5. Leptin increases SK1 expression and enzymatic activity and VEGF expression in BT-549 cells. Figure S6. Leptin activates p-STAT3 in a dose-dependent manner In MCF-7. Figure S7. Leptin does not increase SK1 expression and enzymatic activity and VEGF expression in MCF-7 cells. Figure S8. Leptin activates p-STAT3 in BT-474 cells. Figure S9. JAK2 silencing or PI3K/Akt inhibition does not abrogate STAT3 phosphorylation. Figure S10. STAT3 silencing potentiates SK1 expression. Figure S11. STAT3 siRNA silenced STAT3 and induced SK1 expression. Figure S12. Inhibition of ERK1/2 and SFK modulates STAT3 phosphorylation. Figure S13. ERK silencing increases STAT3 phosphorylation. Figure S14. ERK silencing attenuates SK1 expression and enzymatic activity and VEGF expression. Figure S15. SFK phosphorylation at site 416 is important for leptin signalling. Figure S16. Inhibition of ERK1/2 and SFK modulates STAT3 phosphorylation and attenuates SK1 and VEGF expression. Figure S17. Knockdown of SHP2 decreases SFK phosphorylation. Figure S18. Knockdown of SHP2 decreases SFK phosphorylation and SPHK1 expression. (PDF 2 MB) [file 13058_2014_426_MOESM2_ESM.pdf]

Supplementary figure 1

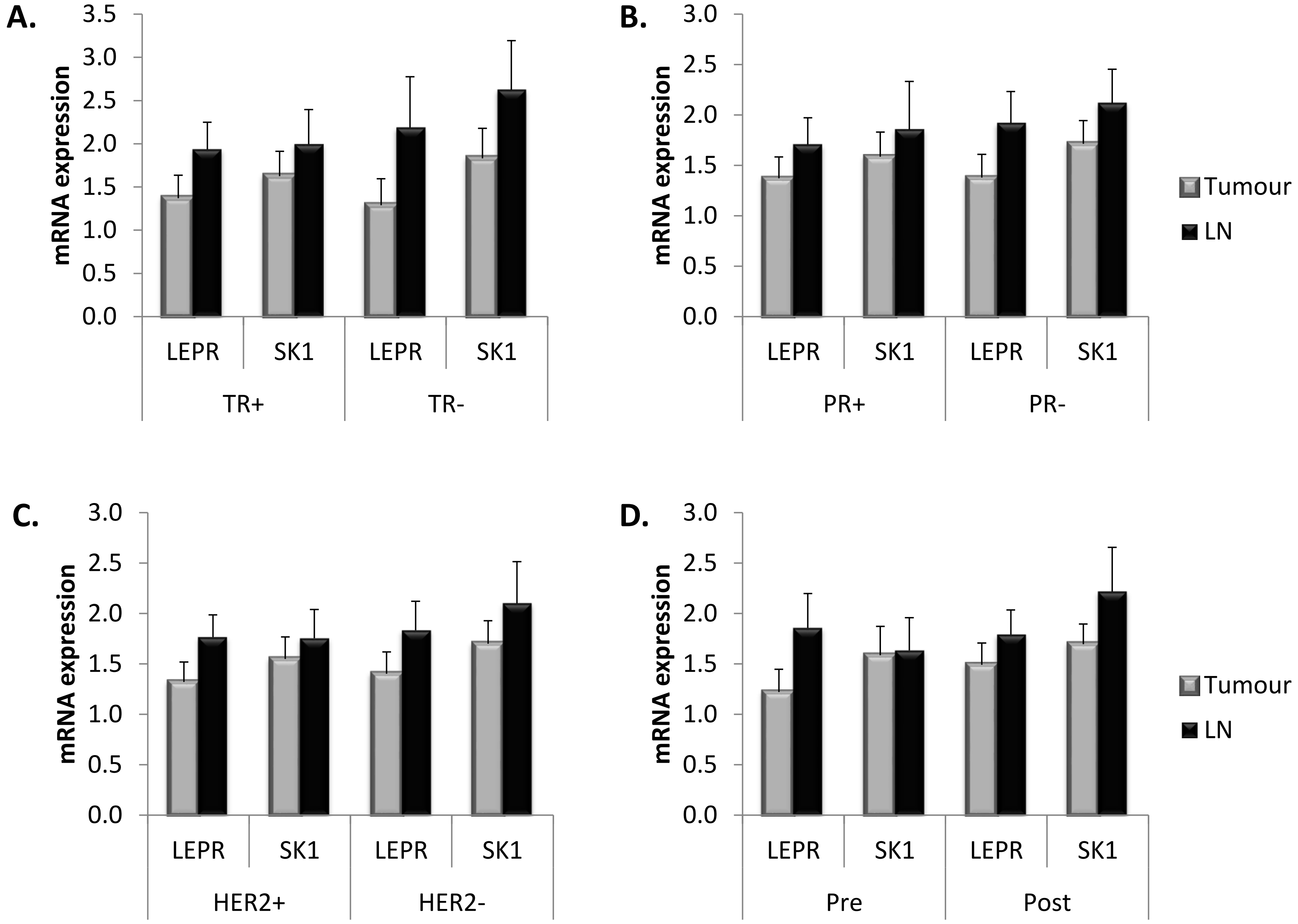

Figure S1. LEPR-Long and SK1 expression is slightly elevated in triple-negative breast cancer patients. RNA was extracted from human breast cancer clinical samples and expression of SK1 and LEPR-Long was determined by qRT-PCR, normalised against (GAPDH, GUSB, TBP, 18S and MRPL19) and analysed using qBase software. Expression of LEPR-Long and SK1 in primary tumours and metastatic LNs of **(A)** TR-positive and negative, **(B)** PR-positive and negative , **(C)** HER2-positive and negative breast cancer patients. **(D)** Expression of LEPR-Long and SK1 with respect to menopausal status (pre- or post- the menopause). *Columns*, represent the mean; *bars*, SEM. (*, P<0.05; **, P<0.01; §, P<0.001; NS, not significant, P>0.05). HER2, human epidermal growth factor receptor 2; LNs, lymph nodes; PR, progesterone-receptor; TR, triple.

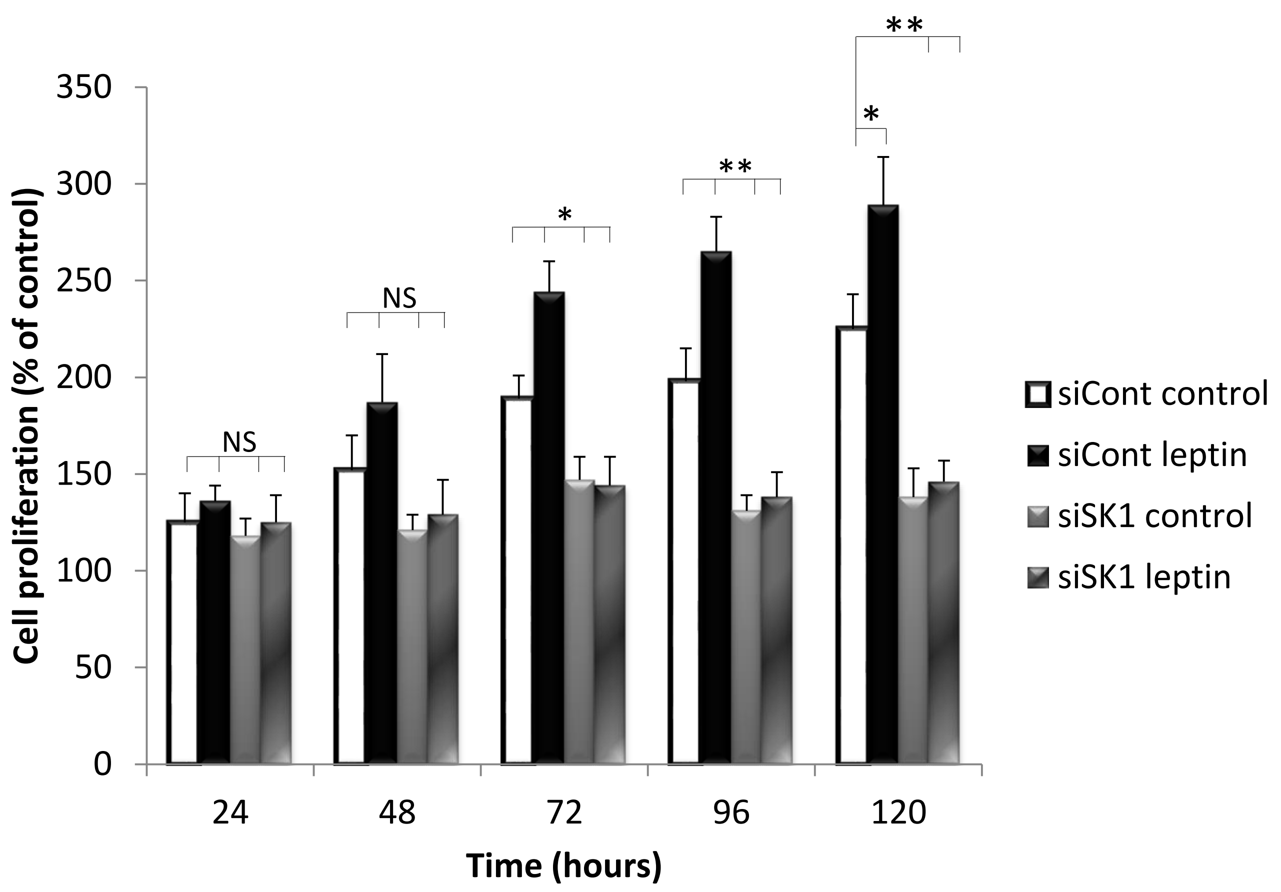

Figure S2. Leptin does not increase the proliferation of BT-549 cells in the absence of SK1 signalling. BT-549 cells were transfected with specific siRNA against SK1 (siSK1) or control siRNA (siCont) using oligofectamine™. Cells then were starved overnight then incubated with 1000 ng/ml leptin for 5 days. Cell proliferation was followed using MTT assay. *Columns*, mean of three independent experiments performed in sextuplicate; *bars*, SEM. (*, $P<0.05$; **, $P<0.01$; §, $P<0.001$; NS, not significant, $P>0.05$).

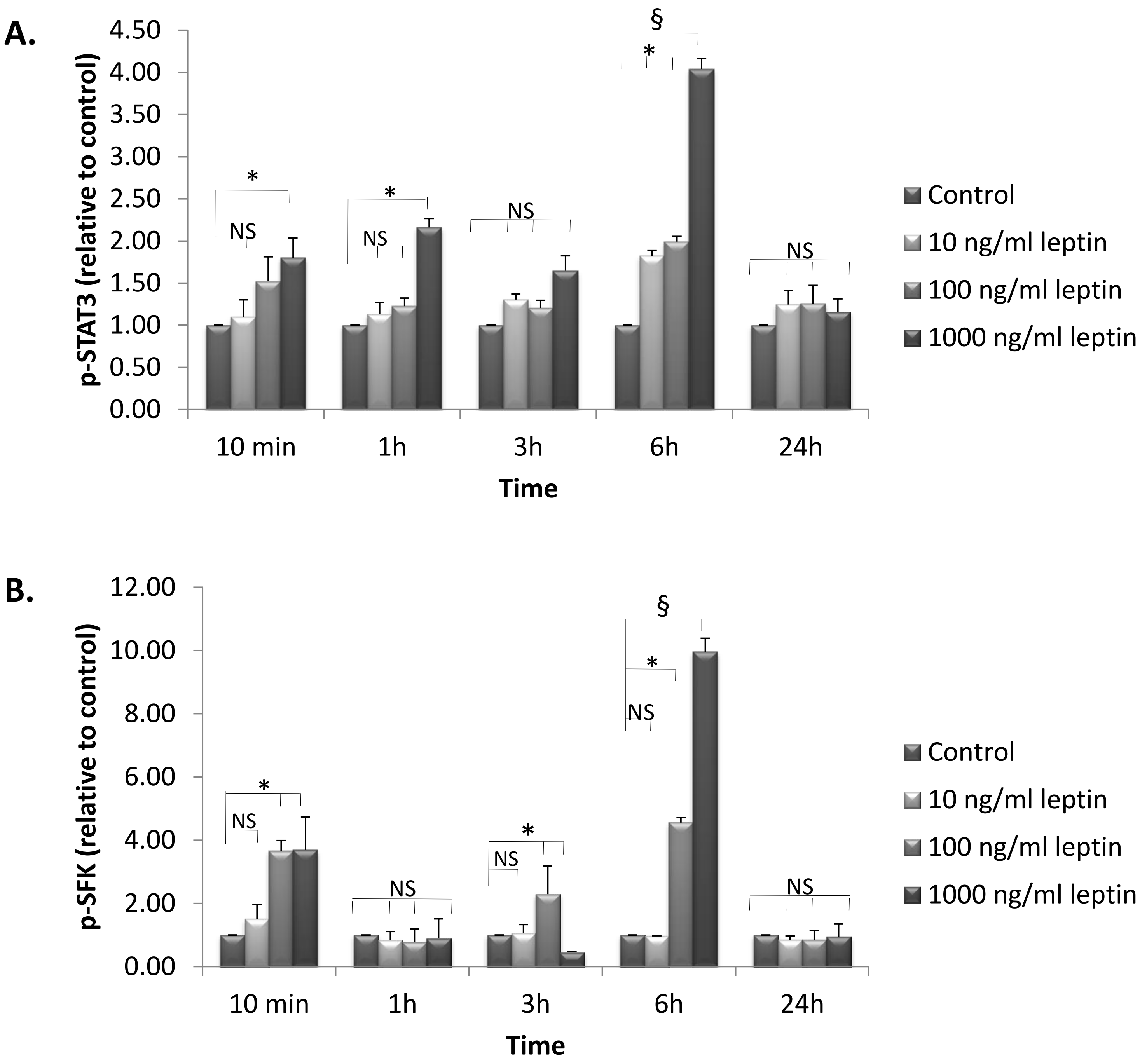

Figure S3. Leptin activates p-STAT3 and P-SFK in MDAMB-231 cells. Densitometric analysis of p-STAT3 **(A)** and p-SFK **(B)** of three independent western blots using Image J software. Levels of p-STAT3 and p-SFK were normalised to GAPDH levels and expressed as fold change relative to control at each time point. Blots are representative of three independent experiments. *Columns*, mean of three independent experiments; *bars*, SEM. (*, $P<0.05$; **, $P<0.01$; §, $P<0.001$; NS, not significant, $P>0.05$).

Supplementary figure 4

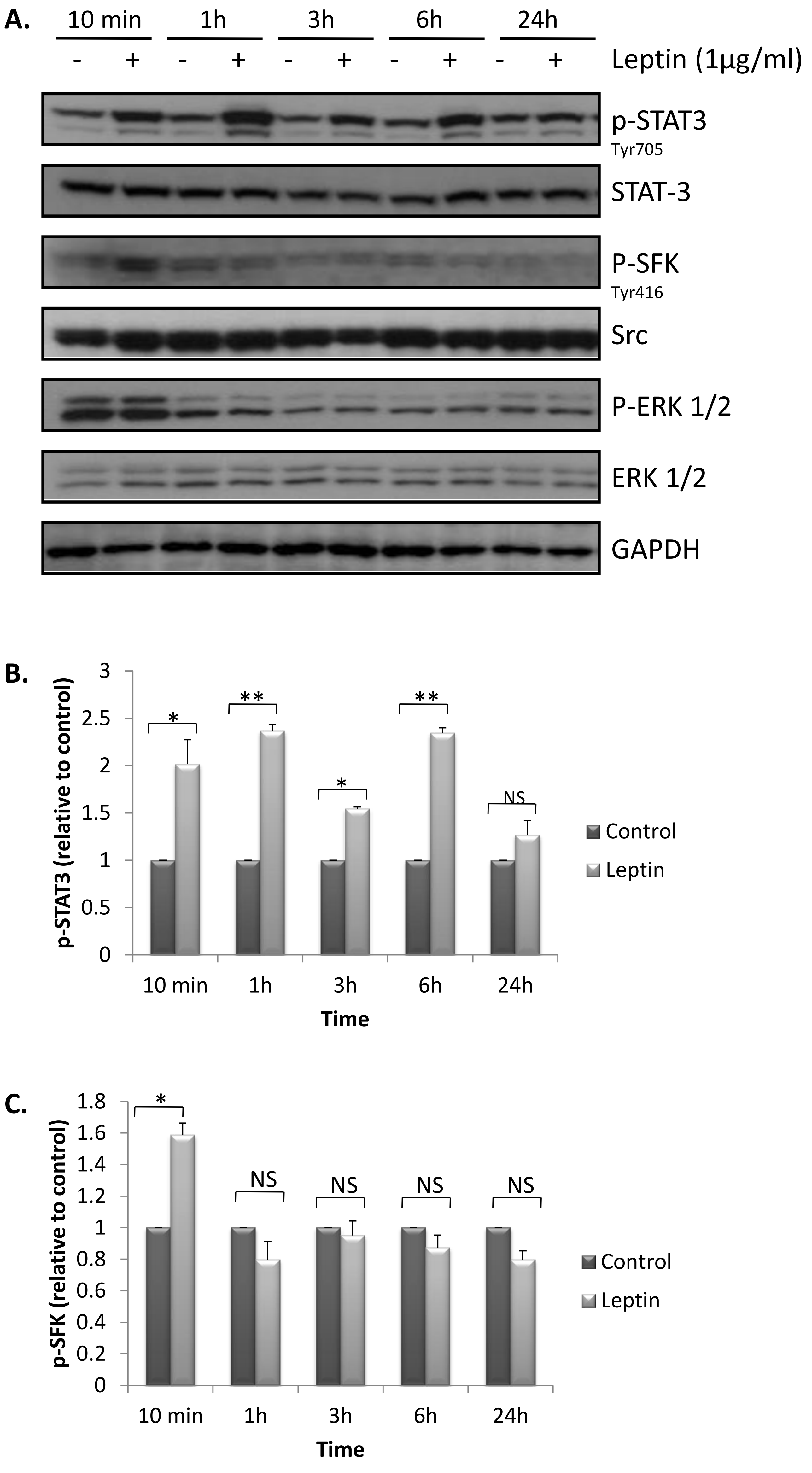

Figure S4. Leptin activates p-STAT3 and P-SFK in BT-549 cells. Cells were starved overnight in serum-free media then exposed to 1000 ng/ml leptin for indicated times. **(A)** Cell lysates obtained after each time point were separated on a 10% SDS-PAGE gel and probed for phosphorylation of STAT3, SFK and ERK1/2. Densitometric analysis of p-STAT3 **(B)** and p-SFK **(C)** levels of three independent western blots using Image J software. Levels of p-STAT3 and p-SFK were normalised to GAPDH levels and expressed as fold change relative to control at each time point. Blots are representative of three independent experiments. *Columns*, mean of three independent experiments; *bars*, SEM. (*, P<0.05; **, P<0.01; §, P<0.001; NS, not significant, P>0.05).

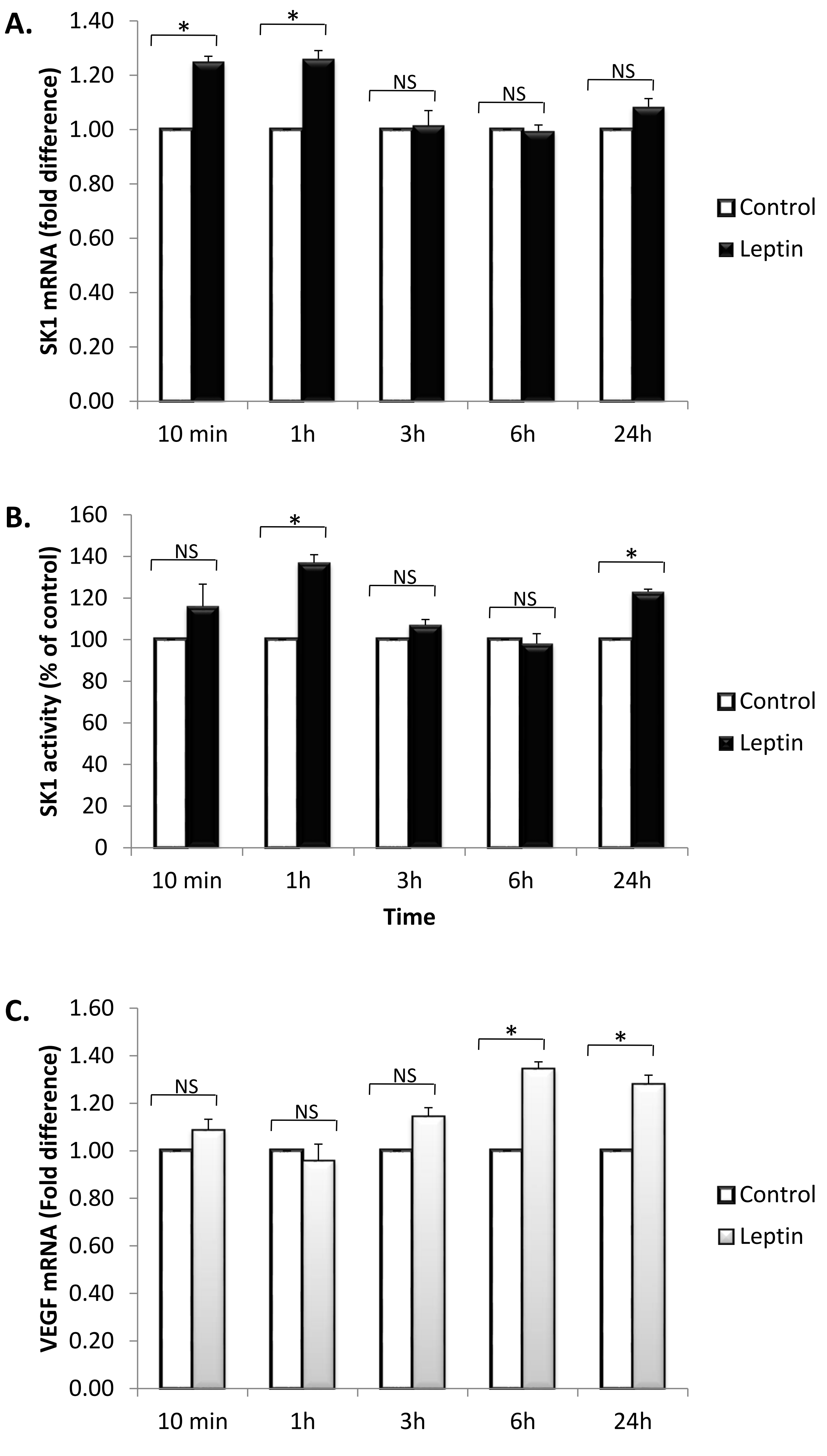

Figure S5. Leptin increases SK1 expression and enzymatic activity and VEGF expression in BT-549 cells. Cells were starved overnight in serum-free media then exposed to 1000 ng/ml leptin for indicated times. SK1 **(A)** and VEGF **(C)** expression and SK1 activity **(B)** were measured in cell lysates containing equal amounts of mRNA and protein. SK1 activity was measured by radiolabelling of sphingosine. For qRT-PCR, SK1 and VEGF were normalised against housekeeping genes (GAPDH, YWHAZ and UBC) and analysed using qBase software. Columns, mean of three independent experiments performed in triplicate; bars, SEM. (*, P<0.05; **, P<0.01; §, P<0.001; NS, not significant, P>0.05) when comparing levels of SK1, and VEGF mRNA to control levels.

Supplementary figure 6

A.

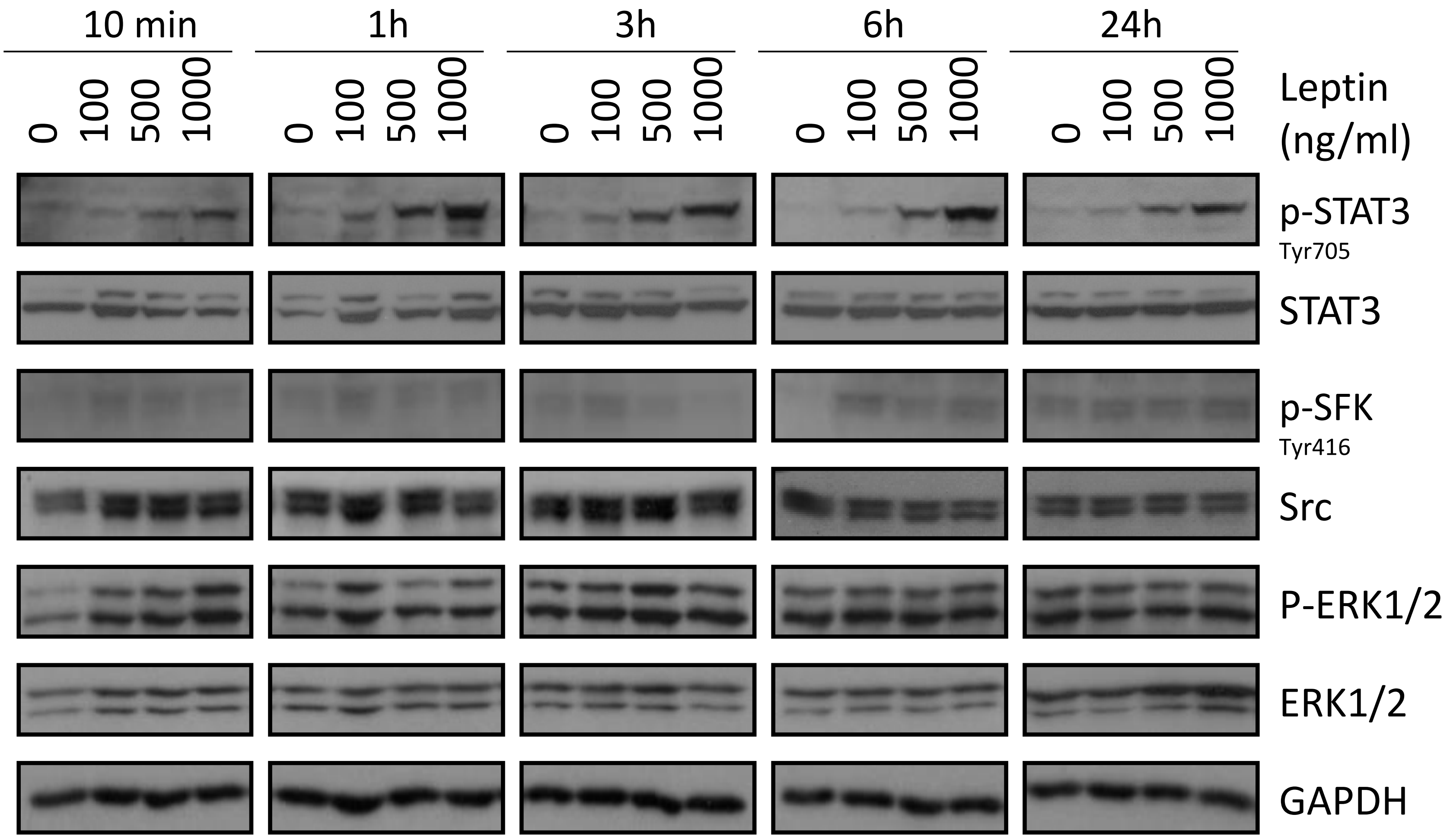

B.

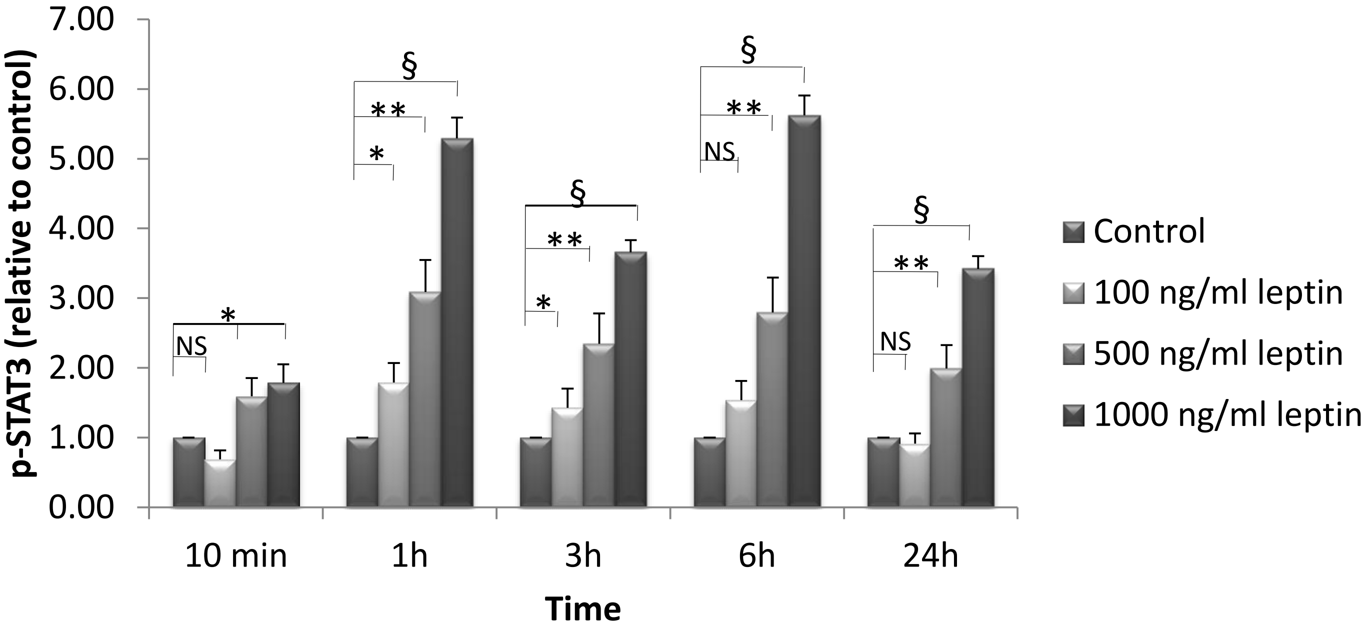

Figure S6. Leptin activates p-STAT3 in a dose-dependent manner In MCF-7. Cells were starved overnight in serum-free media then exposed to 100-1000 ng/ml leptin for indicated times. **(A)** Cell lysates obtained after each time point were separated on a 10% SDS-PAGE gel and probed for phosphorylation of STAT3, SFK and ERK1/2. **(B)** Densitometric analysis of p-STAT3 levels of three independent western blots using Image J software. Levels of p-STAT3 were normalised to GAPDH levels and expressed as fold change relative to control at each time point. Blots are representative of three independent experiments. Columns, mean of three independent experiments; bars, SEM. (*, $P<0.05$; **, $P<0.01$; §, $P<0.001$; NS, not significant, $P>0.05$).

A.

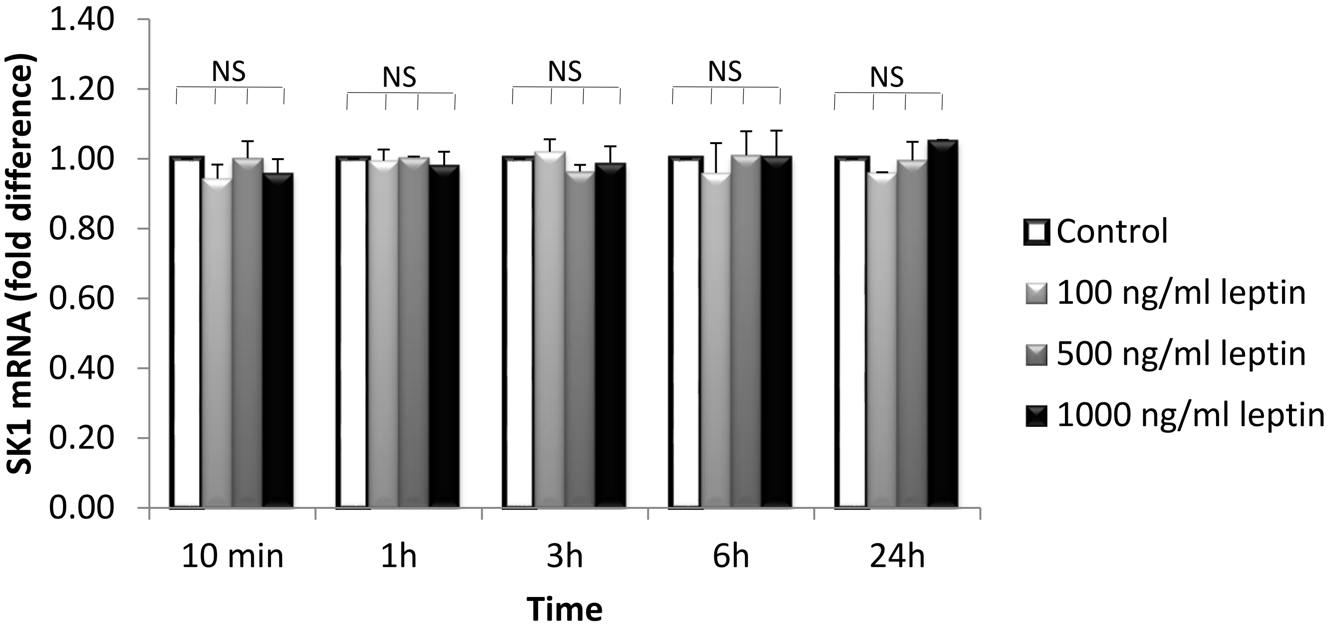

B.

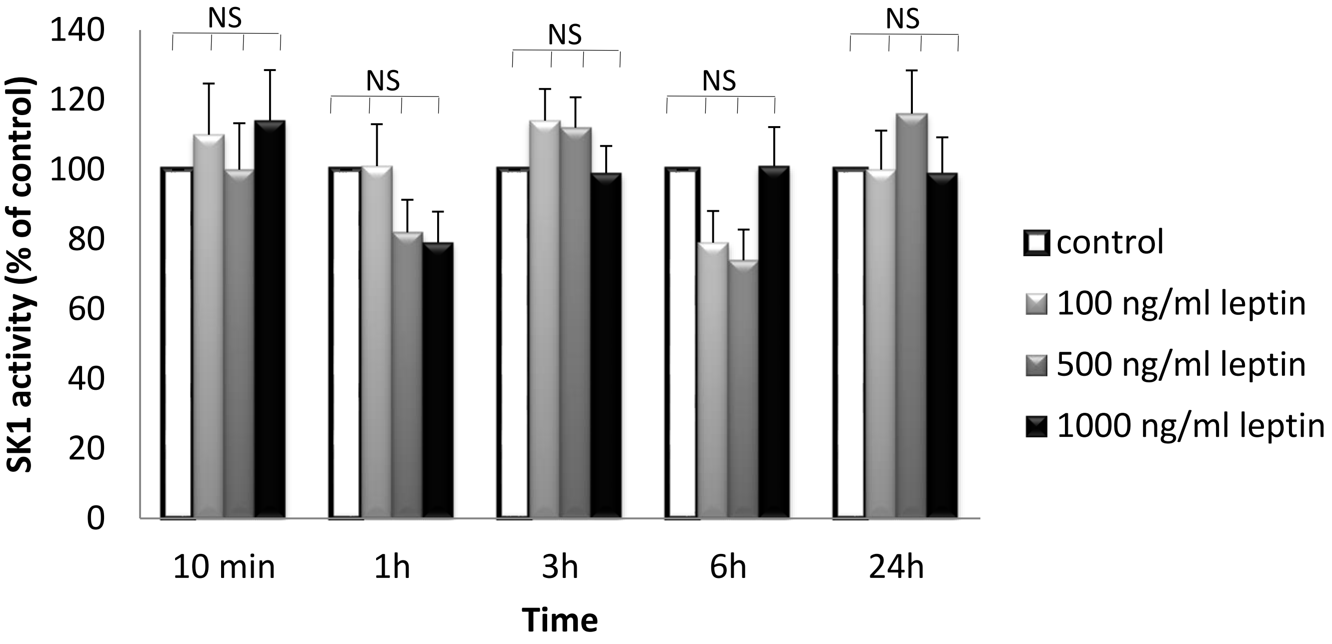

C.

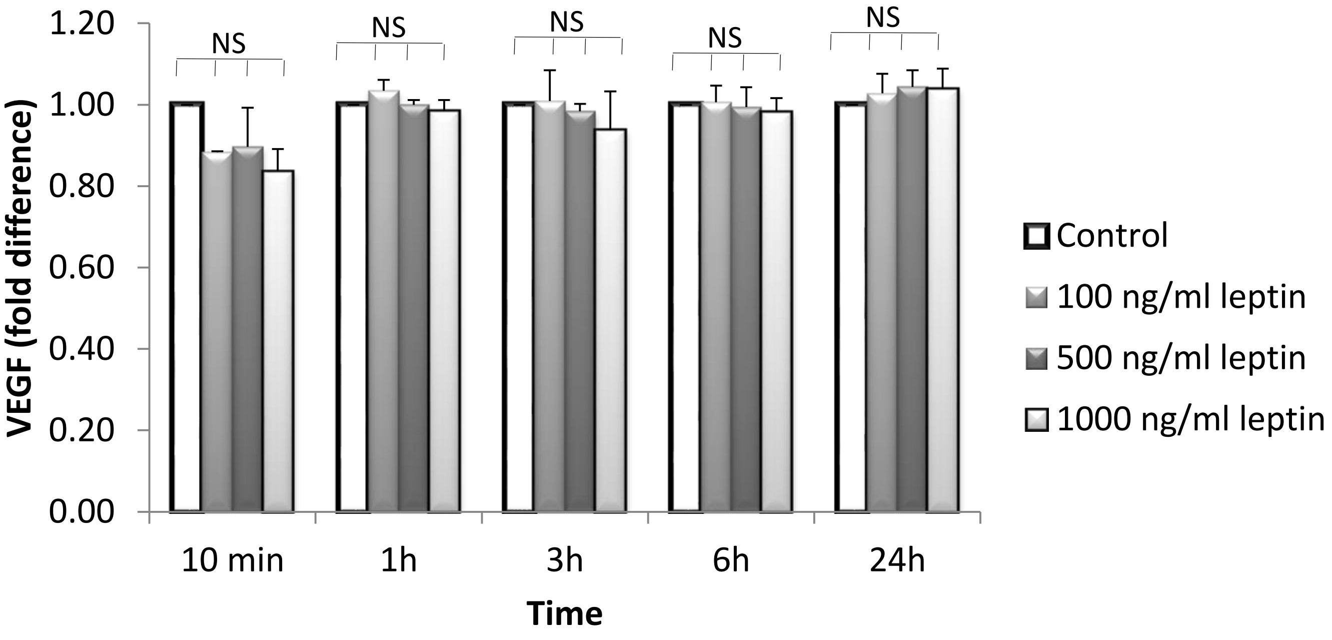

Figure S7. Leptin does not increase SK1 expression and enzymatic activity and VEGF expression in MCF-7 cells. Cells were starved overnight in serum-free media then exposed to 100-1000 ng/ml leptin for indicated times. SK1 **(A)** and VEGF **(C)** expression and SK1 activity **(B)** were measured in cell lysates containing equal amounts of mRNA and protein. SK1 activity was measured by radiolabelling of sphingosine. For qRT-PCR, SK1 and VEGF expression were normalised against housekeeping genes (GAPDH, YWHAZ and UBC) and analysed using qBase software. Columns, mean of three independent experiments; bars, SEM. (*, P<0.05; **, P<0.01; §, P<0.001; NS, not significant, P>0.05).

Supplementary figure 8

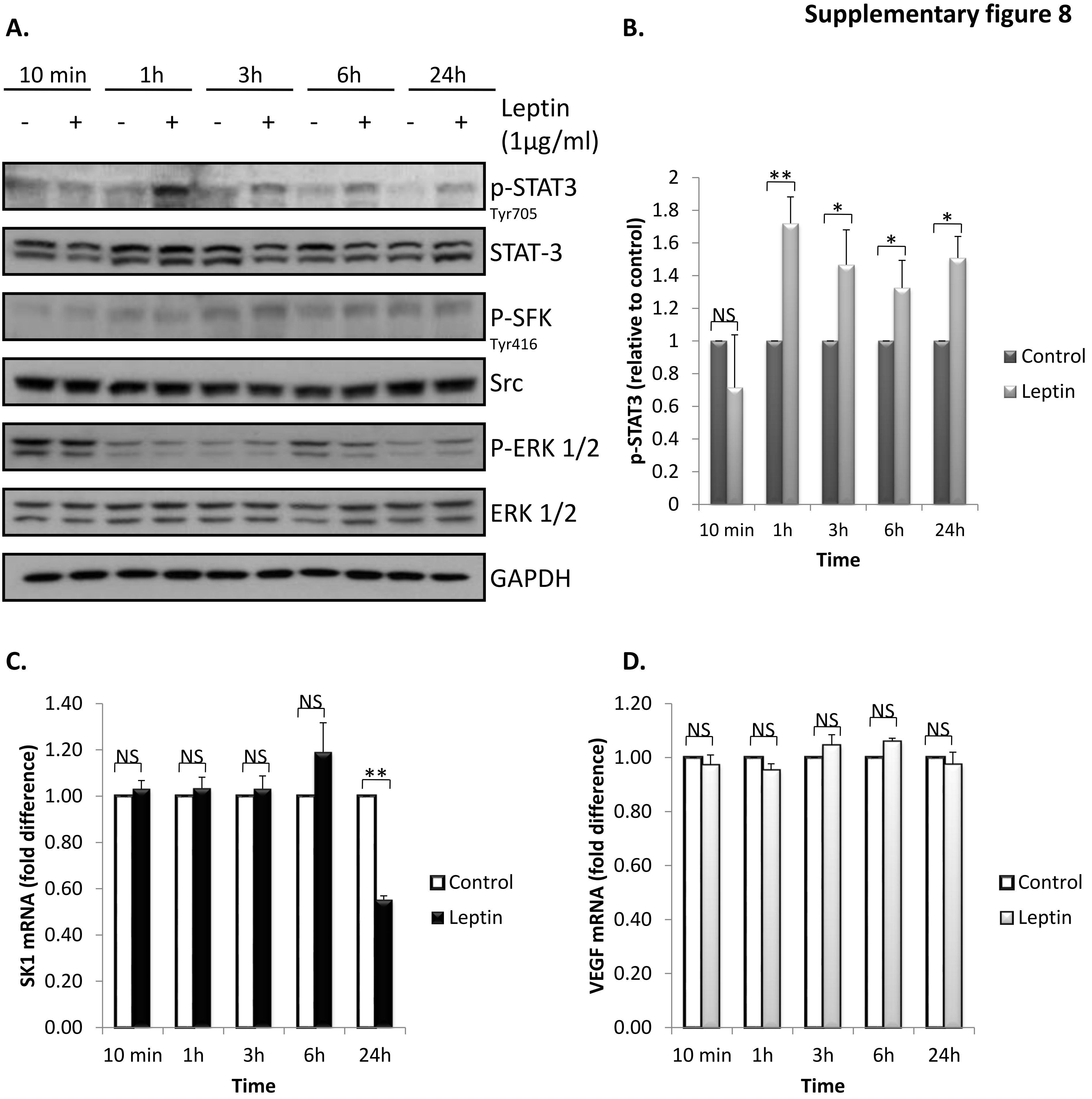

Figure S8. Leptin activates p-STAT3 in BT-474 cells. Cells were starved overnight in serum-free media then exposed to 1000 ng/ml leptin for indicated times. **(A)** Cell lysates obtained after each time point were separated on a 10% SDS-PAGE gel and probed for phosphorylation of STAT3, SFK and ERK1/2. **(B)** Densitometric analysis of p-STAT3 levels of three independent western blots using Image J software. Levels of p-STAT3 were normalised to GAPDH levels and expressed as fold change relative to control at each time point. Blots are representative of three independent experiments. Expression of SK1 **(C)** and VEGF **(D)** determined by qRT-PCR, normalised against housekeeping genes (GAPDH, YWHAZ and UBC) and analysed using qBase software. Columns, mean of three independent experiments performed in triplicate; bars, SEM. (*, $P<0.05$; **, $P<0.01$; §, $P<0.001$; NS, not significant, $P>0.05$).

Supplementary figure 9

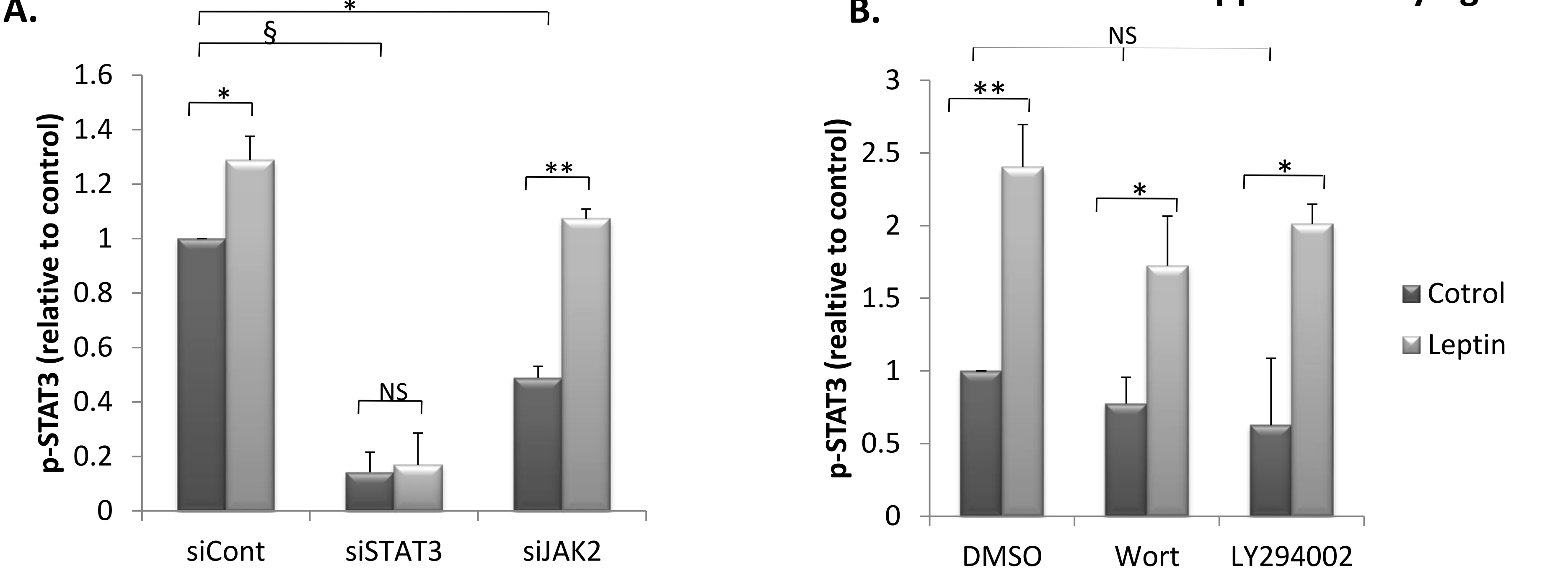

Figure S9. JAK2 silencing or PI3K/Akt inhibition does not abrogate STAT3 phosphorylation. (A) MDAMB-231 cells were transfected with specific siRNA against STAT3 (siSTAT3) and JAK2 (siJAK2) or control siRNA (siCont) using oligofectamine™ (Oligo). Cells were starved overnight in serum-free media then exposed to 1000 ng/ml leptin for 6 h. **(B)** MDAMB-231 cells were starved overnight in serum-free media then pre-treated with PI3K/Akt inhibitors wortmannin (1 µM) and LY294002 (10 µM) for 1 h followed by stimulation with 1000 ng/ml leptin for 6 h**.** Densitometric analysis of p-STAT3 levels **(A-B)** of three independent western blots using Image J software. Levels of p-STAT3 were normalised to GAPDH levels and expressed as fold change relative to siCont. *Columns*, mean of three independent experiments; *bars*, SEM. (*, P<0.05; **, P<0.01; §, P<0.001; NS, not significant, P>0.05).

Supplementary figure 10

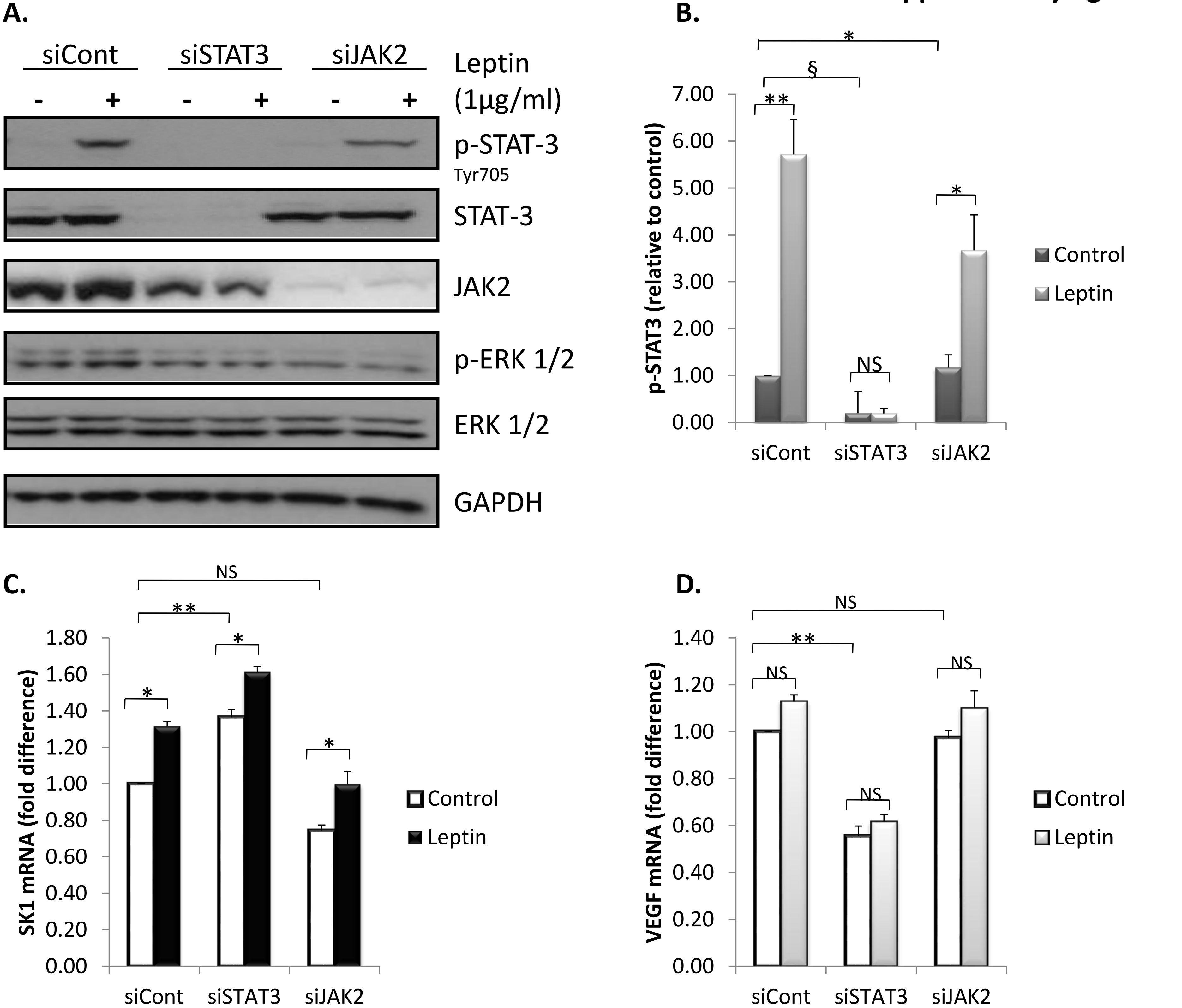

Figure S10. STAT3 silencing potentiates SK1 expression. BT-549 cells were transfected with specific siRNA against STAT3 (siSTAT3) and JAK2 (siJAK2) or control siRNA (siCont) using oligofectamine™ (Oligo). Cells were starved overnight in serum-free media then exposed to 1000 ng/ml leptin for 1 h **(A, B)**. **(A)** Cell lysates obtained were separated on a 10% SDS-PAGE gel and probed for phosphorylation of STAT3, JAK2 and ERK1/2 to verify knockdown efficiency. **(B)** Densitometric analysis of p-STAT3 levels of three independent western blots using Image J software. Levels of p-STAT3 were normalised to GAPDH levels and expressed as fold change relative to siCont. Blots are representative of three independent experiments. Expression of SK1 **(C)** and VEGF **(D)** determined by qRT-PCR, normalised against housekeeping genes (GAPDH, YWHAZ and UBC) and analysed using qBase software. *Columns*, mean of three independent experiments performed in triplicate; *bars*, SEM. (*, P<0.05; **, P<0.01; §, P<0.001; NS, not significant, P>0.05).

Supplementary figure 11

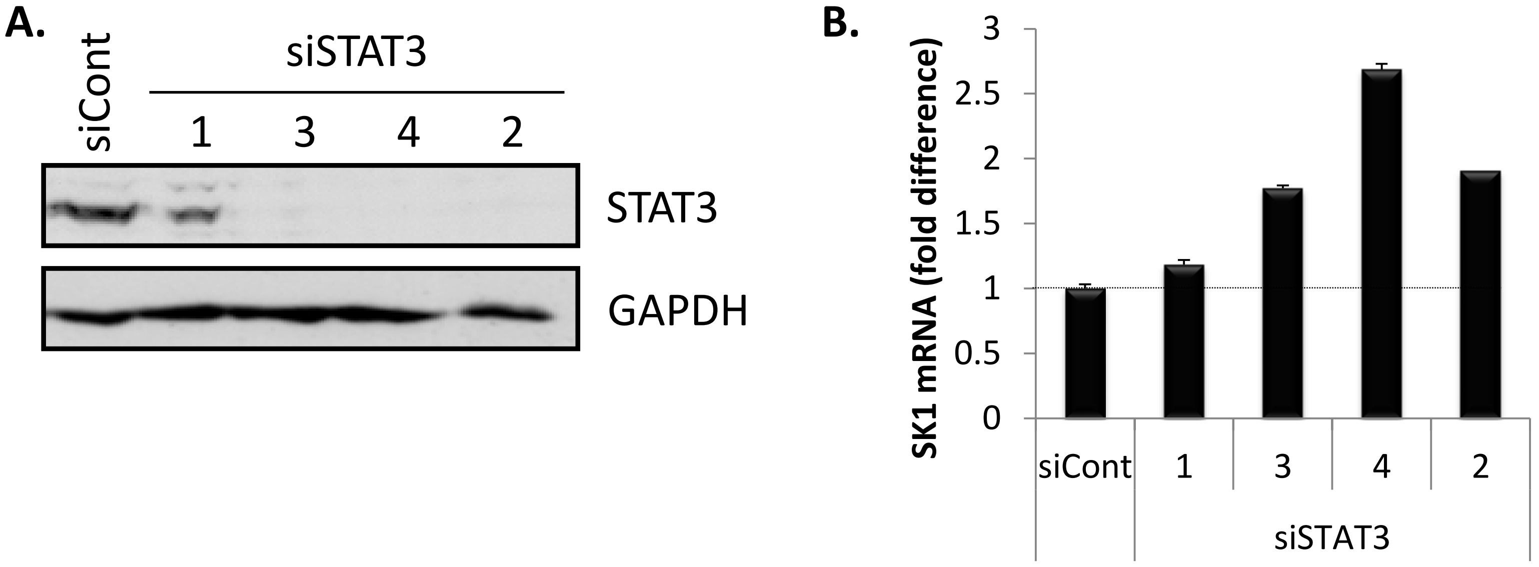

Figure S11. STAT3 siRNA silenced STAT3 and induced SK1 expression. MDAMB-231 cells were transfected with specific siRNA against each of STAT3 (siSTAT3) individual pool or control siRNA (siCont) using oligofectamine™. **(A)** Cell lysates obtained were separated on a 10% SDS-PAGE gel and probed for phosphorylation of STAT3 to verify knockdown efficiency. **(B)** Expression of SK1 determined by qRT-PCR, normalised against housekeeping genes (GAPDH, YWHAZ and UBC) and analysed using qBase software. *Columns*, mean of three independent experiments performed in triplicate; *bars*, SEM.

Supplementary figure 12

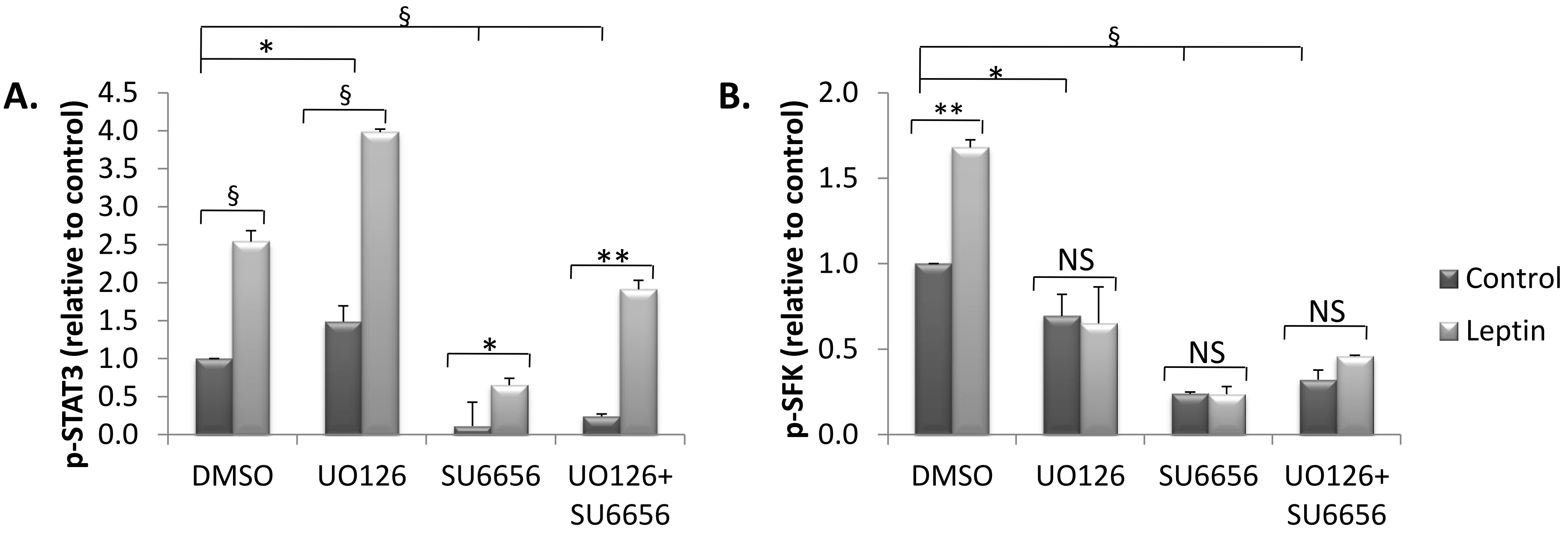

Figure S12. Inhibition of ERK1/2 and SFK modulates STAT3 phosphorylation. MDAMB-231 cells were starved overnight in serum-free media and pre-treated with MEK1/2 inhibitor U0126 (10 µM) and/or SFK inhibitor SU6656 (10 µM) for 1 h followed by stimulation with 1000 ng/ml leptin for 6 h. Densitometric analysis of p-STAT3 **(A)** and p-SFK **(B)** levels of three independent experiments using Image J software. Levels of p-STAT3 and p-SFK were normalised to GAPDH levels and expressed as fold change relative to DMSO. Blots are representative of three independent experiments. *Columns*, mean of three independent experiments; *bars*, SEM. (*, P<0.05; **, P<0.01; §, P<0.001; NS, not significant, P>0.05).

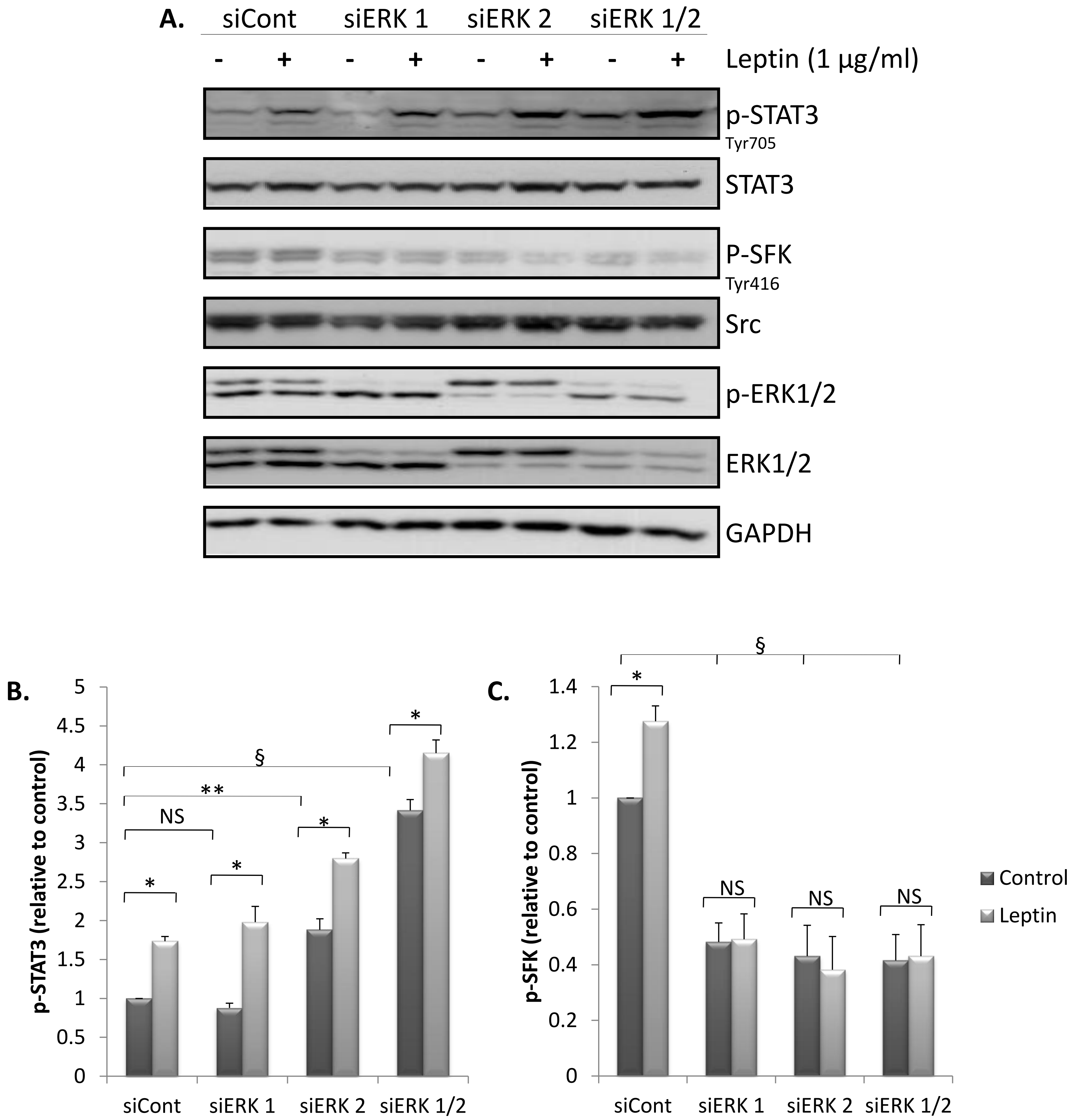

Figure S13. ERK silencing increases STAT3 phosphorylation. MDAMB-231 cells were transfected with specific siRNA against ERK1 (siERK1), ERK2 (siERK2) and ERK1/2 (siERK1/2) or control siRNA (siCont) using oligofectamine^{TM} (Oligo). Cells were starved overnight in serum-free media then exposed to 1000 ng/ml leptin for 6 h. **(A)** Cell lysates were separated on a 10% SDS-PAGE gel and probed for phosphorylation of STAT3, SFK and ERK1/2 to verify knockdown efficiency. Densitometric analysis of p-STAT3 **(B)** and p-SFK **(C)** levels of three independent western blots using Image J software. Levels of p-STAT3 and p-SFK were normalised to GAPDH levels and expressed as fold change relative to siCont. Blots are representative of three independent experiments. *Columns*, mean of three independent experiments; *bars*, SEM. (*, P<0.05; **, P<0.01; §, P<0.001; NS, not significant, P>0.05).

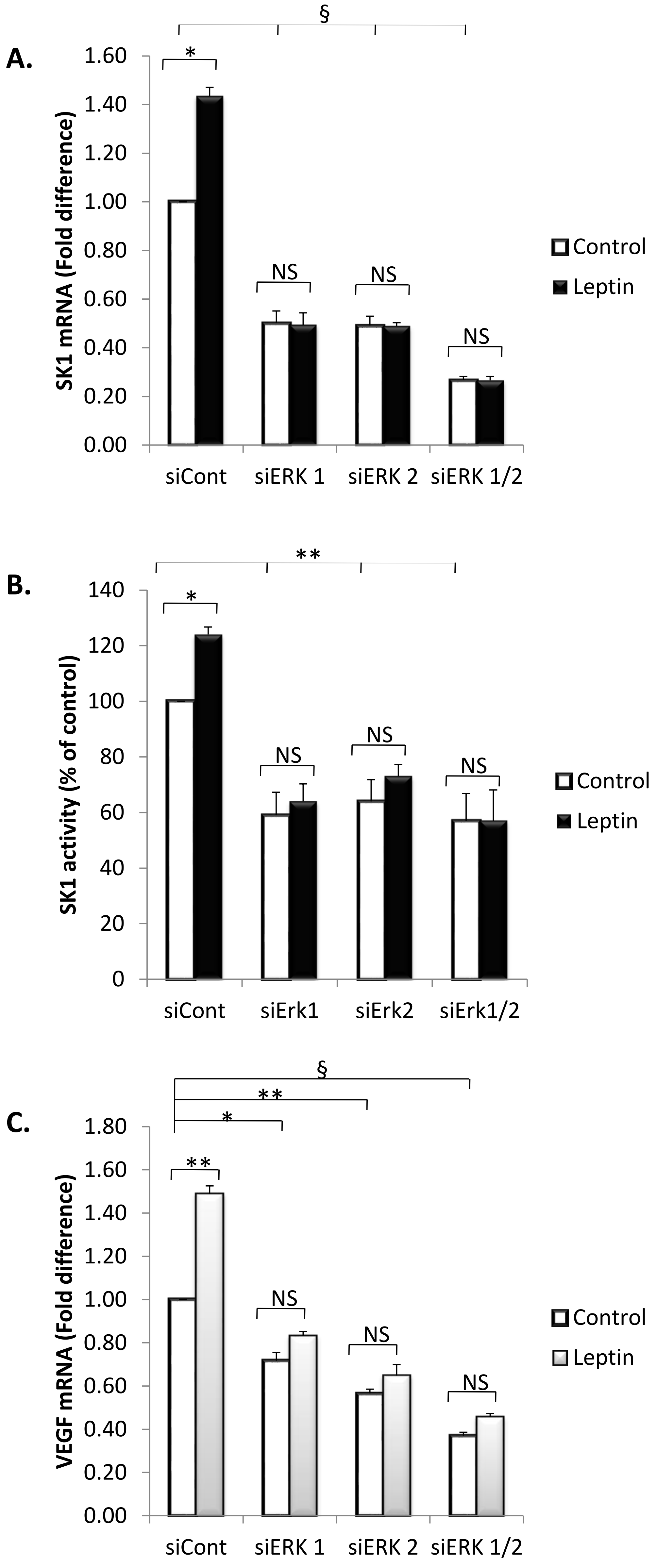

Figure S14. ERK silencing attenuates SK1 expression and enzymatic activity and VEGF expression. MDAMB-231 cells were transfected with specific siRNA against ERK1 (siERK1), ERK2 (siERK2) and ERK1/2 (siERK1/2) or control siRNA (siCont) using oligofectamine^{TM} (Oligo). Cells were starved overnight in serum-free media then exposed to 1000 ng/ml leptin for 6 h. SK1 **(A)** and VEGF **(C)** expression and SK1 activity **(B)** were measured in cell lysates containing equal amounts of mRNA and protein. SK1 activity was measured by radiolabelling of sphingosine. For qRT-PCR, SK1 and VEGF were normalised against housekeeping genes (GAPDH, YWHAZ and UBC) and analysed using qBase software. *Columns*, mean of three independent experiments performed in triplicate; *bars*, SEM. (*, P<0.05; **, P<0.01; §, P<0.001; NS, not significant, P>0.05).

Supplementary figure 15

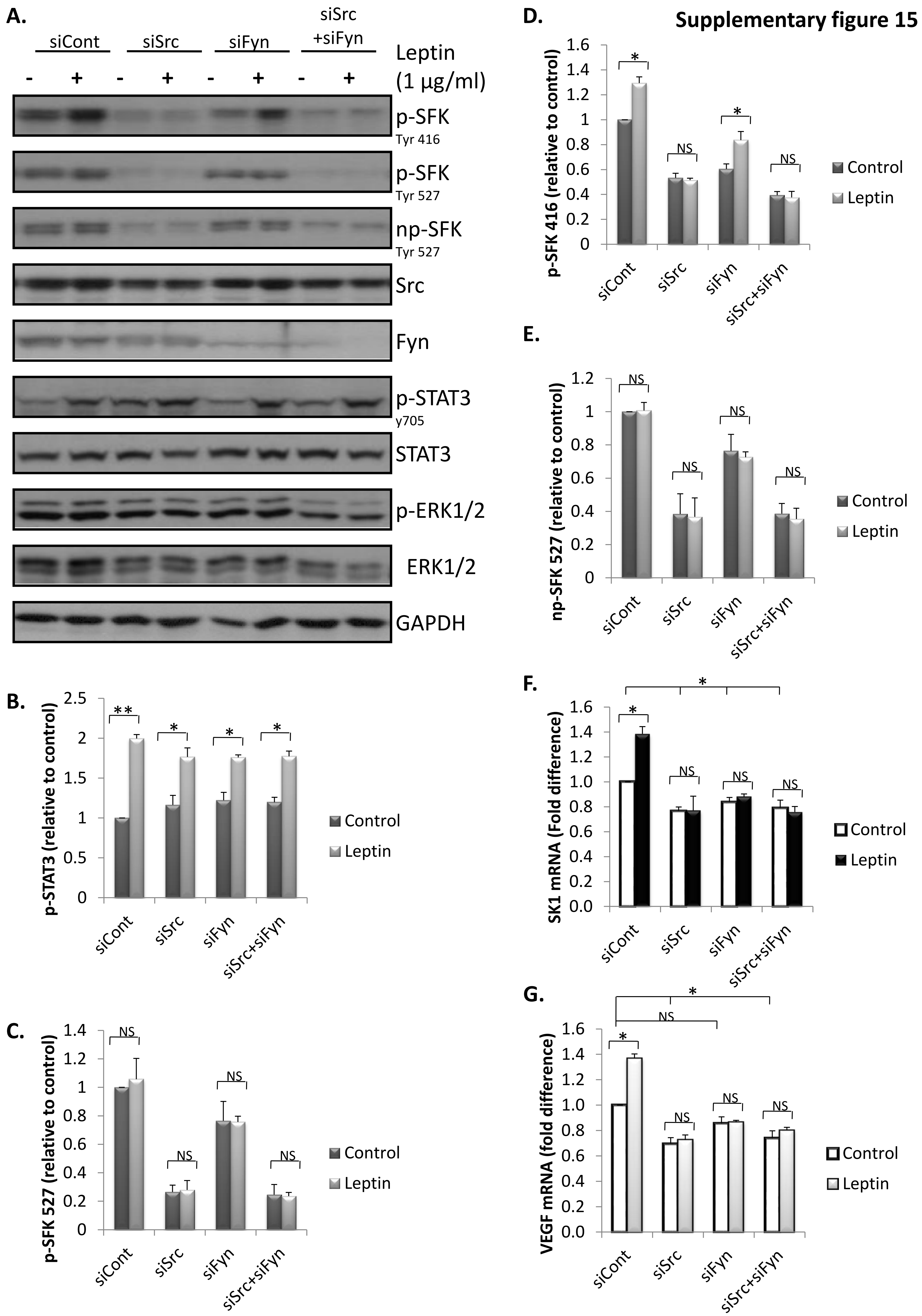

Figure S15. SFK phosphorylation at site 416 is important for leptin signalling. MDAMB-231 cells were transfected with specific siRNA against Src (siSrc), Fyn (siFyn), combination or control siRNA (siCont) using oligofectamineTM. Cells were starved overnight in serum-free media then exposed to 1000 ng/ml leptin for 6 h. **(A)** Cell lysates were separated on a 10% SDS-PAGE gel and probed for phosphorylation of STAT3, SFK-416, SFK-527, non-phospho SFK-527 and ERK1/2. Densitometric analysis of p-STAT3 **(B)** p-SFK 416 **(C)** p-SFK527 **(D)** and np-SFK 527 **(E)** levels of three independent experiments using Image J software. Levels of p-STAT3 and p-SFK at the different sites were normalised to GAPDH levels and expressed as fold change relative to siCont. Blots are representative of three independent experiments. SK1 **(F)** and VEGF **(G)** expression were measured in cell lysates containing equal amounts of mRNA. For qRT-PCR, SK1 and VEGF were normalised against housekeeping genes (GAPDH, YWHAZ and UBC) and analysed using qBase software. *Columns*, mean of three independent experiments performed in triplicate; *bars*, SEM. (*, P<0.05; **, P<0.01; §, P<0.001; NS, not significant, P>0.05).

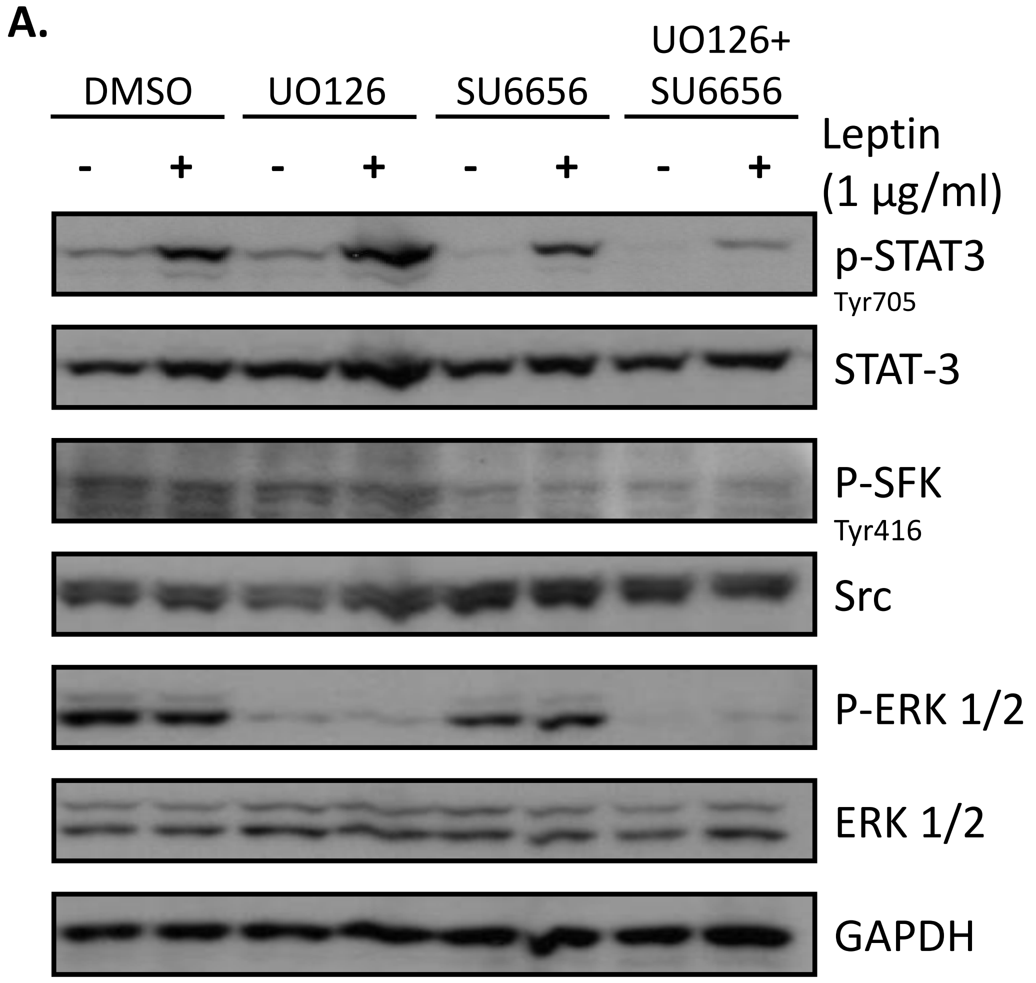

Figure S16. Inhibition of ERK1/2 and SFK modulates STAT3 phosphorylation and attenuates SK1 and VEGF expression. BT-549 cells were starved overnight in serum-free media and pre-treated with MEK 1/2 inhibitor U0126 (10 µM) and/or SFK inhibitor SU6656 (10 µM) for 1 h followed by stimulation with 1000 ng/ml leptin for 1 h. **(A)** Cell lysates were separated on 10% SDS-PAGE gel and probed for phosphorylation of STAT3, SFK and ERK1/2. Densitometric analysis of p-STAT3 **(B)** and p-SFK **(C)** levels of three independent experiments using Image J software. Levels of p-STAT3 and p-SFK were normalised to GAPDH levels and expressed as fold change relative to DMSO. Blots are representative of three independent experiments. Expression of SK1 **(D)** and VEGF **(E)** was measured in cell lysates containing equal amounts of mRNA. For qRT-PCR, SK1 and VEGF were normalised against housekeeping genes (GAPDH, YWHAZ and UBC) and analysed using qBase software. *Columns*, mean of three independent experiments performed in triplicate; *bars*, SEM. (*, $P<0.05$; **, $P<0.01$; §, $P<0.001$; NS, not significant, $P>0.05$).

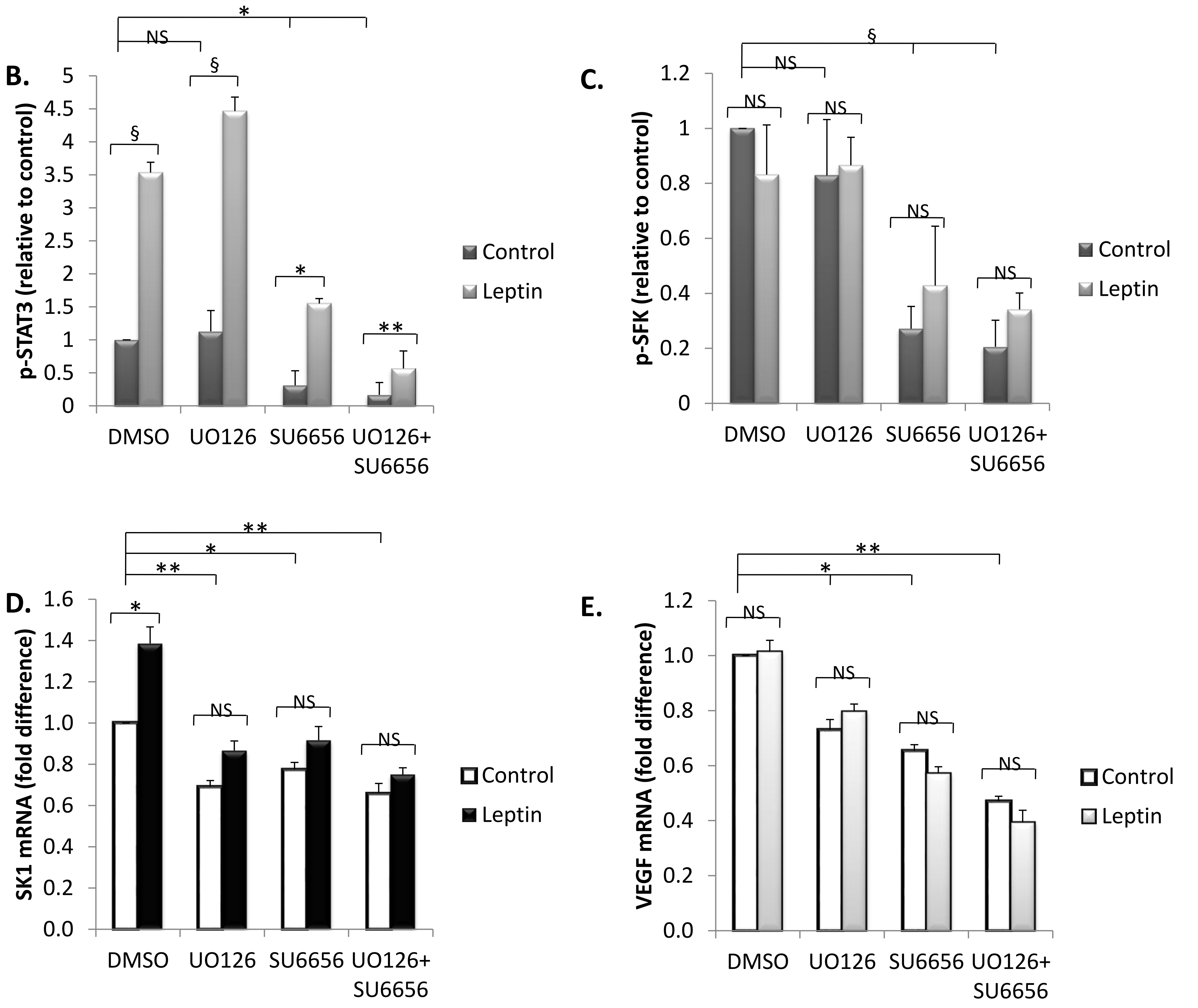

Supplementary figure 17

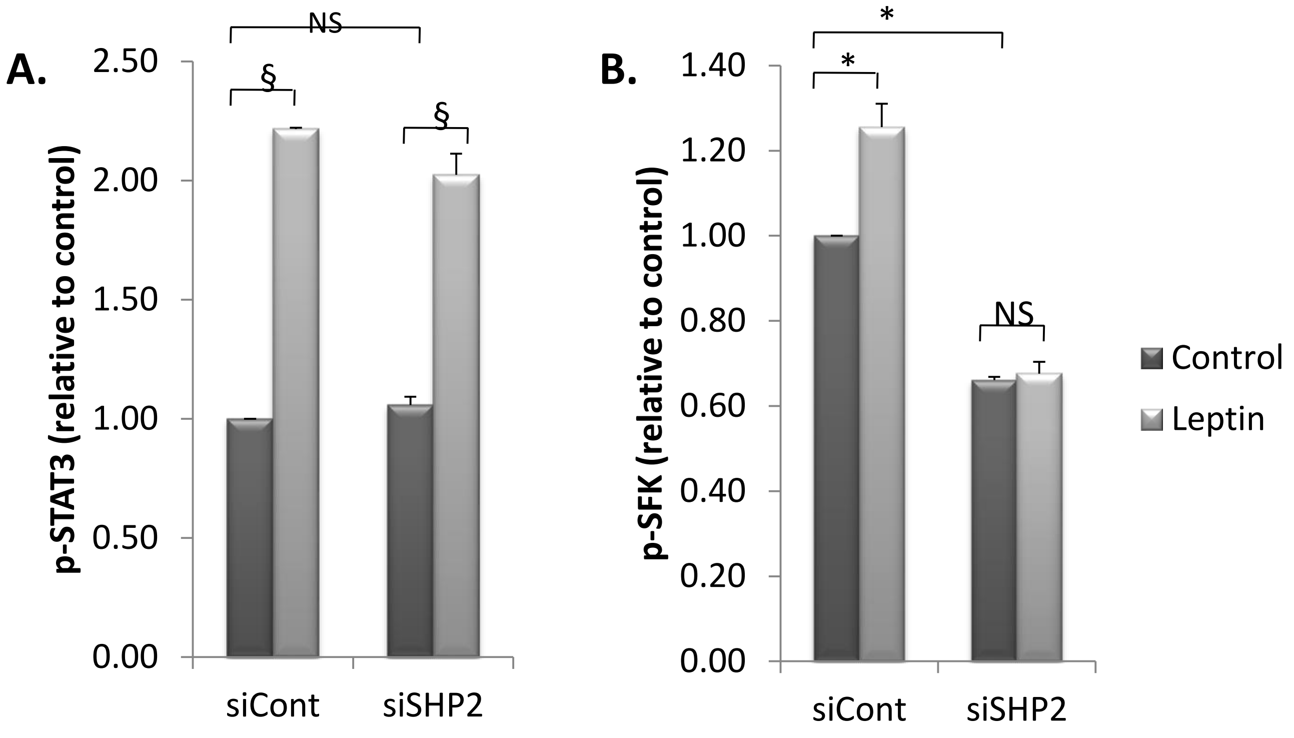

Figure S17. Knockdown of SHP2 decreases SFK phosphorylation. MDAMB-231 cells were transfected with specific siRNA against SHP2 (siSHP2) or control siRNA (siCont) using oligofectamine^{TM}. Then cells were starved overnight in serum-free media followed by stimulation with 1000 ng/ml leptin for 6 h. Densitometric analysis of p-STAT3 **(A)** and p-SFK **(B)** levels of three independent western blots using Image J software. Levels of p-STAT3 and p-SFK were normalised to GAPDH levels and expressed as fold change relative to siCont. *Columns*, mean of three independent experiments performed in triplicate; *bars*, SEM. (*, P<0.05; **, P<0.01; §, P<0.001; NS, not significant, P>0.05).

Supplementary figure 18

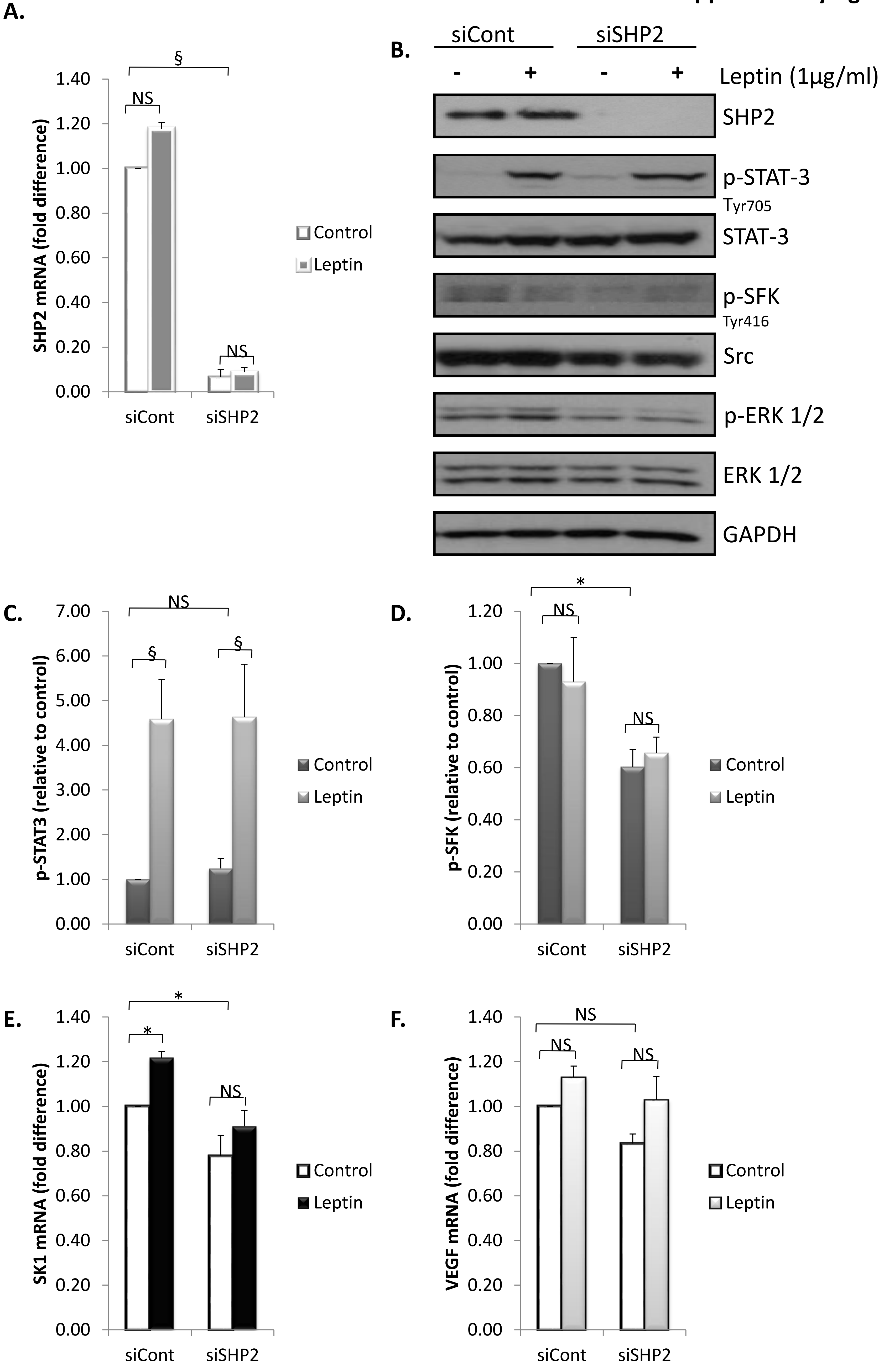

Figure S18. Knockdown of SHP2 decreases SFK phosphorylation and SPHK1 expression. BT-549 cells were transfected with specific siRNA against SHP2 (siSHP2) or control siRNA (siCont). Then cells were starved overnight in serum-free media followed by stimulation with 1 μg/ml leptin for 6 h. **A.** Expression of SHP2 determined by qRT-PCR to verify knockdown efficiency. **B.** Cell lysates obtained were separated on a 10% SDS-PAGE gel and probed for phosphorylation of STAT3, SFK and ERK1/2. Blots are representative of three independent experiments. Densitometric analysis of p-STAT3 **(C)** and p-SFK **(D)** levels of three independent western blots using Image J software. Levels of p-STAT3 and p-SFK were normalised to GAPDH levels and expressed as fold change relative to siCont. Expression of SPHK1 **(E)** and VEGF **(F)** mRNA was determined by qRT-PCR, normalised against housekeeping genes (GAPDH, YWHAZ and UBC) and analysed using qBase software. *Columns*, mean of three independent experiments performed in triplicate; *bars*, SEM. (*, $P<0.05$; **, $P<0.01$; §, $P<0.001$; NS, not significant, $P>0.05$).
