# Supplementary figures and images for: Leptin induces upregulation of sphingosine kinase 1 in oestrogen receptor-negative breast cancer via Src family kinase-mediated, janus kinase 2-independent pathway
Source: Breast Cancer Res. 2014 Oct 25;16:426. doi: 10.1186/s13058-014-0426-6 (PMC4303110; doi:10.1186/s13058-014-0426-6)

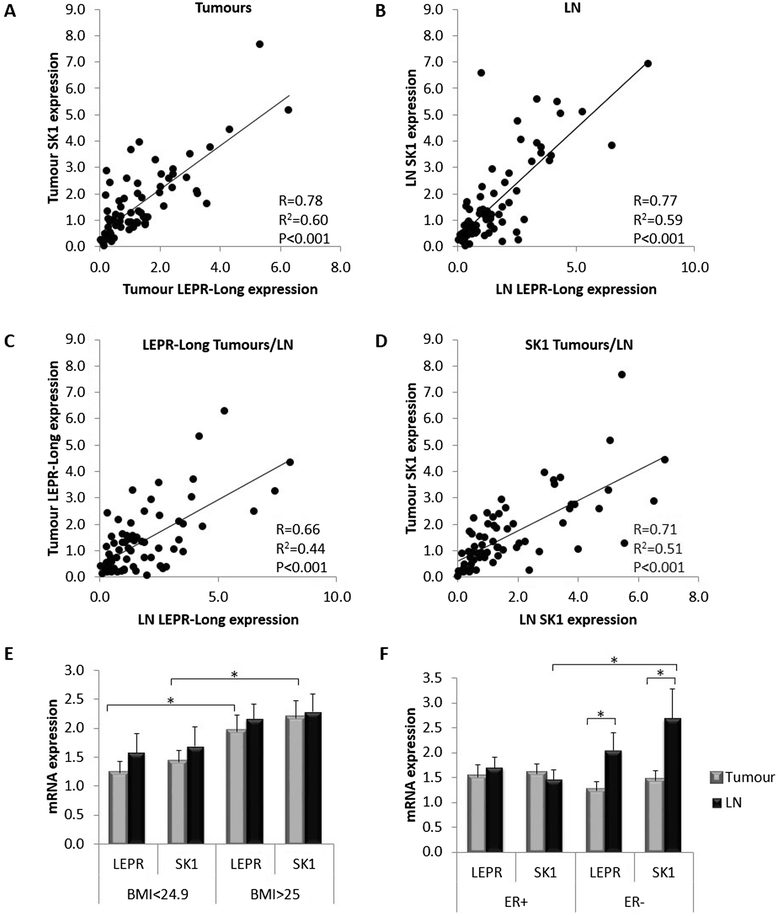

Supplement: Supplementary file 3 — Authors’ original file for figure 1 [file 13058_2014_426_MOESM3_ESM.gif]

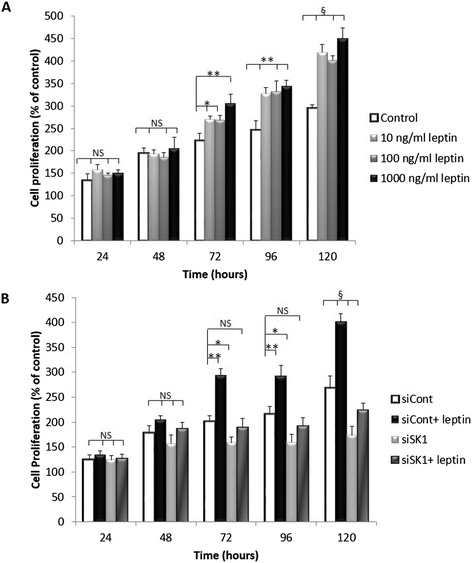

Supplement: Supplementary file 4 — Authors’ original file for figure 2 [file 13058_2014_426_MOESM4_ESM.gif]

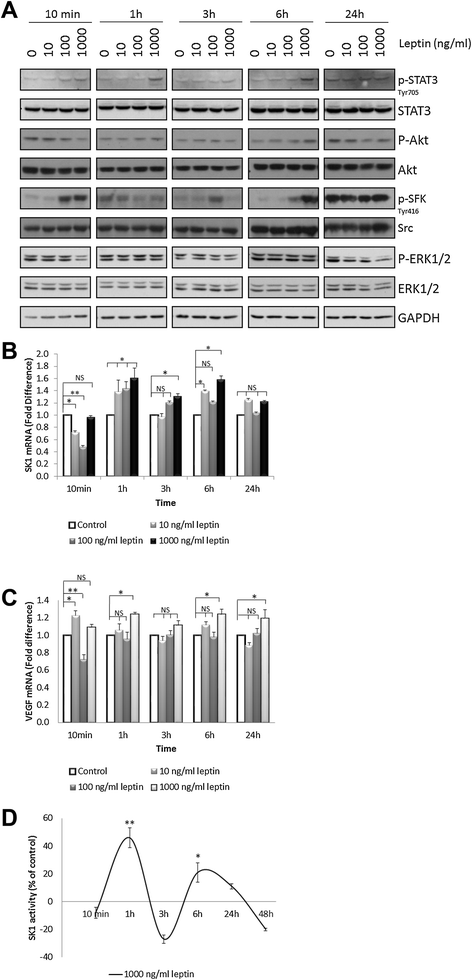

Supplement: Supplementary file 5 — Authors’ original file for figure 3 [file 13058_2014_426_MOESM5_ESM.gif]

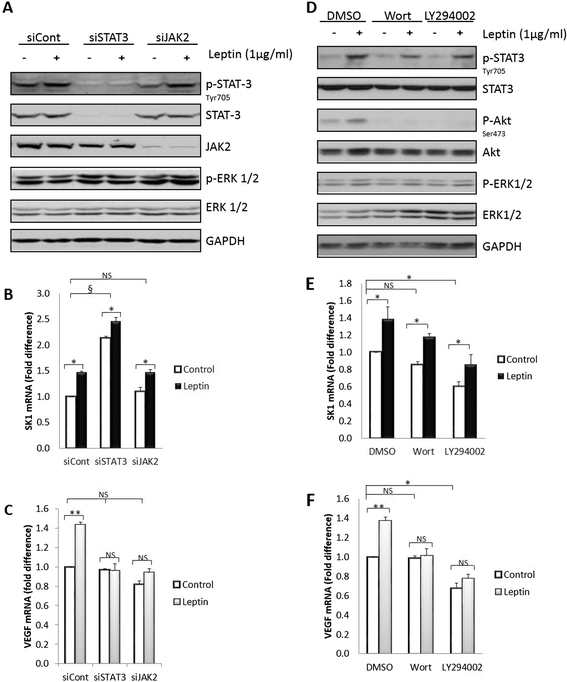

Supplement: Supplementary file 6 — Authors’ original file for figure 4 [file 13058_2014_426_MOESM6_ESM.gif]

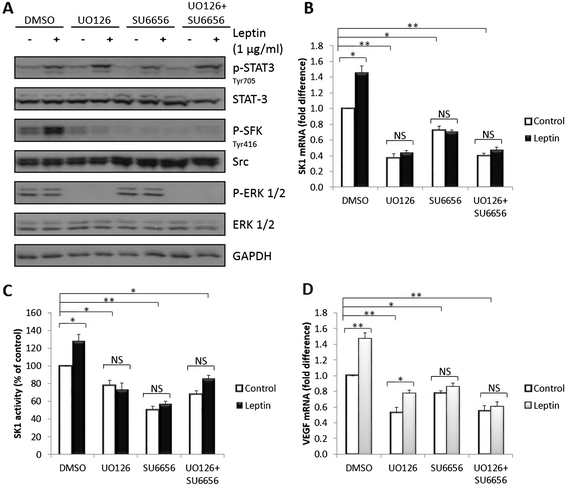

Supplement: Supplementary file 7 — Authors’ original file for figure 5 [file 13058_2014_426_MOESM7_ESM.gif]

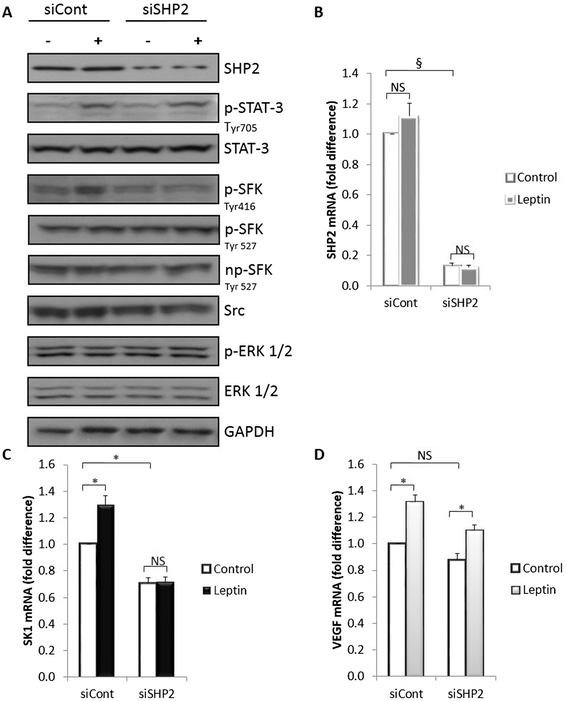

Supplement: Supplementary file 8 — Authors’ original file for figure 6 [file 13058_2014_426_MOESM8_ESM.gif]

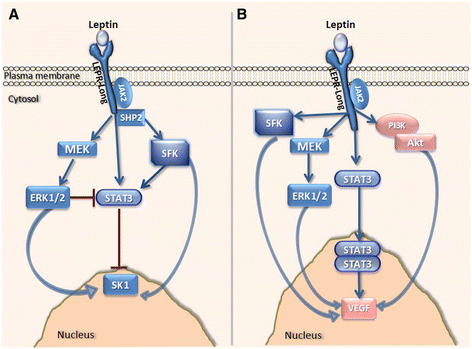

Supplement: Supplementary file 9 — Authors’ original file for figure 7 [file 13058_2014_426_MOESM9_ESM.gif]
